# Exploring viral neuropathic pain: Molecular mechanisms and therapeutic implications

**DOI:** 10.1371/journal.ppat.1012397

**Published:** 2024-08-08

**Authors:** Songchao Xu, Huili Li, Zhangran Ai, Ruijuan Guo, Hao Cheng, Yun Wang

**Affiliations:** 1 Department of Anesthesiology, Beijing Friendship Hospital, Capital Medical University, Beijing, China; 2 Department of Anesthesiology, Beijing Ditan Hospital, Capital Medical University, Beijing, China; University of Alberta, CANADA

## Abstract

As the Coronavirus Disease 2019 (COVID-19) pandemic continues, there is a growing concern regarding the relationship between viral infections and neuropathic pain. Chronic neuropathic pain resulting from virus-induced neural dysfunction has emerged as a significant issue currently faced. However, the molecular mechanisms underlying this phenomenon remain unclear, and clinical treatment outcomes are often suboptimal. Therefore, delving into the relationship between viral infections and neuropathic pain, exploring the pathophysiological characteristics and molecular mechanisms of different viral pain models, can contribute to the discovery of potential therapeutic targets and methods, thereby enhancing pain relief and improving the quality of life for patients. This review focuses on HIV-related neuropathic pain (HNP), postherpetic neuralgia (PHN), and neuropathic pain caused by Severe Acute Respiratory Syndrome Coronavirus 2 (SARS-CoV-2) infections, examining rodent models and relevant cellular molecular pathways. Through elucidating the connection between viral infections and neuropathic pain, it aims to delineate the current limitations and challenges faced by treatments, thereby providing insights and directions for future clinical practice and research.

## Introduction

In recent years, viral infections have posed persistent challenges to global public health, manifesting as both acute illnesses and chronic conditions with enduring consequences.

Neuropathic pain is caused by a lesion or disease of the somatosensory nervous system [1], closely associated with glial cell activation [2], ion channel alterations [3], and neuroinflammation [4]. Noteworthy among these are HIV/AIDS, herpes zoster (HZ), and the novel coronavirus, each introducing distinctive neuropathic pain that significantly impacts afflicted individuals. HIV/AIDS, with ongoing transmission in countries worldwide, affects nearly 40 million individuals globally [5]. HIV-related neuropathies have become a notable medical challenge in recent years due to the poor penetration of antiretroviral drugs across the blood–brain barrier (BBB) [6]. Approximately 60% of patients experience chronic pain, with neuropathic pain being the most common symptom [7]. Typical manifestations include gradually progressing bilateral numbness, burning sensations, and stabbing pains [8,9]. The reactivation of varicella zoster virus (VZV) leads to herpes zoster, typically manifesting as acute pain and itching in the area of the rash [10]. Although the rash and associated pain are usually self-limiting, a considerable number of patients subsequently develop persistent chronic pain—known as postherpetic neuralgia (PHN) [11]. It refers to pain persisting for at least 4 weeks after clinical healing of the herpes zoster rash, with an incidence ranging from 9% to 34% [12]. Furthermore, in individuals aged 70 and above, the incidence of PHN exceeds 50% [13]. PHN typically characterized by sensations such as burning, electric shocks, stabbing, needle-like pricks, or tearing, accompanied by localized skin hypersensitivity, often leading to emotional, sleep, and quality of life impairments [13]. Similarly, the Severe Acute Respiratory Syndrome Coronavirus 2 (SARS-CoV-2) is widely spreading globally [14]. Although the majority of patients can fully recover, some may develop persistent or new symptoms after the onset of the disease. The World Health Organization refers to these symptoms, which persist for at least 2 months and cannot be explained by other diagnoses, as long COVID syndrome [15]. Studies have indicated that 20% of individuals experiencing post-COVID pain meet the criteria for neuropathic pain symptoms [16]. This type of pain may limit daily activities, affect sleep quality, and lead to psychological health issues such as anxiety and depression [17]. However, at present, the therapeutic efficacy for virus-induced neuropathic pain remains suboptimal.

Due to the large number of affected individuals and the lack of effective treatment options, the global burden of virus-induced neuropathic pain is substantial. These pain symptoms significantly impair patients’ quality of life and place considerable pressure on global healthcare systems and socioeconomic conditions. Therefore, it is imperative to explore preclinical models and molecular mechanisms of these complications and investigate viable treatment approaches. In the following sections, we will review the neurobiological characteristics of the 3 aforementioned viruses (**S1 Table**), focusing on the molecular mechanisms of virus-induced neuropathic pain models, and discuss future research prospects.

## Neurobiological characteristics of different viruses

HIV is an RNA virus, typically appearing circular or oval in shape [18]. Gp120 is the main pathogenic protein. The virus can enter the nervous system through the BBB, where one pathway involves the binding of the gp120 protein on the surface of HIV to the CD4 receptor and C-C chemokine receptors on the surface of brain endothelial cells [19]. Another pathway involves the migration of infected cells, such as monocytes and lymphocytes [20], which are immune cells infected by HIV and may enter brain tissue through the bloodstream, settle there, and release the virus. In contrast to the herpes zoster virus, neuronal damage caused by HIV infection does not rely on direct viral destruction of nerve cells, as HIV lacks the capability to directly infect cells without CD4 receptors [19], such as spinal neurons, dorsal root ganglia (DRG), or Schwann cells. But infection of neuroimmune cells such as microglia and astrocytes in the nervous system is being brought into the limelight [20]. These infected host cells not only synthesize and release HIV viral proteins but also secrete inflammatory mediators, which may lead to neuronal damage, synaptic changes, and further inflammation indirectly through immune stimulation of uninfected astrocytes and microglia [21]. HIV-related neuronal damage is caused by the excessive activation of NMDA-coupled ion channels by excitotoxic substances such as gp120, glutamate, and cellular inflammatory factors [22]. Additionally, viral proteins secreted by infected cells, including Tat, VPR, and Nef, also contribute to neuronal toxicity and apoptosis [23]. Abnormal activation of excitatory synapses may lead to aberrant signal transmission between neurons, resulting in an increase or excessive transmission of pain signals in the nervous system.

VZV is a neurotropic herpesvirus. The surface glycoproteins E and I are key proteins involved in viral cell entry [24]. The virus infects nearly all humans and causes varicella (primary infection) and herpes zoster (reactivation infection) [25]. VZV primarily spreads through respiratory droplets. Initially, the virus typically infects skin and mucosal cells, carrying the virus to resident T cells located in draining lymph nodes [26]. Infected T cells are induced to express skin-homing molecules and transport the virus to dermal fibroblasts and keratinocytes, leading to the characteristic vesicles of varicella [27]. Concurrently, the virus begins to replicate and spread, entering sensory nerve endings, then retrogradely infecting nerve ganglia along the nerve axis, especially the spinal DRG [28]. After recovery from varicella, the virus does not disappear but establishes latent infection in cranial nerve ganglia, DRG, and autonomic ganglia along the entire nerve axis [29]. This latent state is not pathogenic, but in older adults or immunocompromised individuals, latent VZV may reactivate. The mechanisms underlying the persistence of pain after virus reactivation are complex and multifaceted. Firstly, reactivation of the virus infects neurons in the ganglia, and viral replication leads to chronic inflammation and damage to neurons, resulting in increased neuronal excitability and abnormal sensitivity to pain stimuli [28]. Secondly, viral infection may induce structural and functional changes within ganglion cells, affecting the normal signaling mechanisms of neurons [28]. Additionally, viral infection may trigger the activation of immune cells and the release of inflammatory mediators, further exacerbating the inflammatory response of neurons [25]. In the dorsal horn of the spinal cord, the terminals of the sensory neurons release neurotransmitters, which activate secondary neurons [28]. These secondary neurons then transmit the signals to the thalamus. The thalamus acts as a relay station, sending the signals to the cerebral cortex, where they are ultimately perceived as pain [13]. Persistent PHN may also be influenced by changes in the central nervous system, such as abnormally activated pain signaling pathways and damage to pain modulation systems.

SARS-CoV-2 is an enveloped single-stranded RNA virus belonging to the family Coronaviridae, genus β-coronavirus, subgenus Sarbecovirus [30]. The S and E proteins are the main pathogenic proteins [31]. Among them, the S protein can bind to cells expressing the ACE2 receptor, mediating the virus entry into host cells [32]. Despite the multiple protective systems of the central nervous system, some viruses can invade neurons and neuroglial cells and produce neurotoxicity. The virus may enter the central nervous system through the bloodstream, entering the blood circulation during infection and penetrating the brain tissue through the vascular wall, or entering the brain by infecting vascular endothelial cells [33]. This mode allows the virus to directly infect various parts of the central nervous system, including the brain and spinal cord. The virus can also enter the central nervous system through retrograde neuronal transmission, meaning the virus can spread along peripheral nerves to ganglia, then through ganglion axons, bypassing the BBB, and directly entering the central nervous system [34]. Some studies suggest that the virus can also enter the central nervous system through the olfactory nerve pathway, which involves virus infection of olfactory epithelial cells, then entering the olfactory bulb, thereby entering the central nervous system [35]. Autopsy results of patients show brain tissue congestion and edema, and partial neuronal degeneration [36]. The high expression of ACE2 receptors in the spinal dorsal horn may contribute to the virus’s neuroinvasive potential, leading to neuronal dysfunction and synaptic degeneration. Infection and inflammation may activate neuroglial cells, participating in the inflammation and immune response of the nervous system [37]. These neuropathophysiological changes may be closely related to neurological symptoms and complications in patients.

Indeed, various viruses can induce neuropathic pain through diverse neurobiological pathways. HIV infects neuroimmune cells such as microglia and astrocytes, leading to inflammation and neuronal injury [21]. Viral proteins like gp120 activate NMDA receptor and release excitotoxic substances such as glutamate, resulting in abnormal neuronal excitation and increased transmission of pain signals [22]. Reactivation of VZV triggers neuroinflammatory responses and neuronal damage, predominantly contributing to heightened excitability [29]. Meanwhile, SARS-CoV-2 infection causes inflammation and dysfunction of neurons, potentially leading to neurophysiological alterations [37]. However, the molecular mechanisms related to these viruses still require further exploration.

## The main viral neuropathic pain models and potential mechanisms

**Fig 1:**
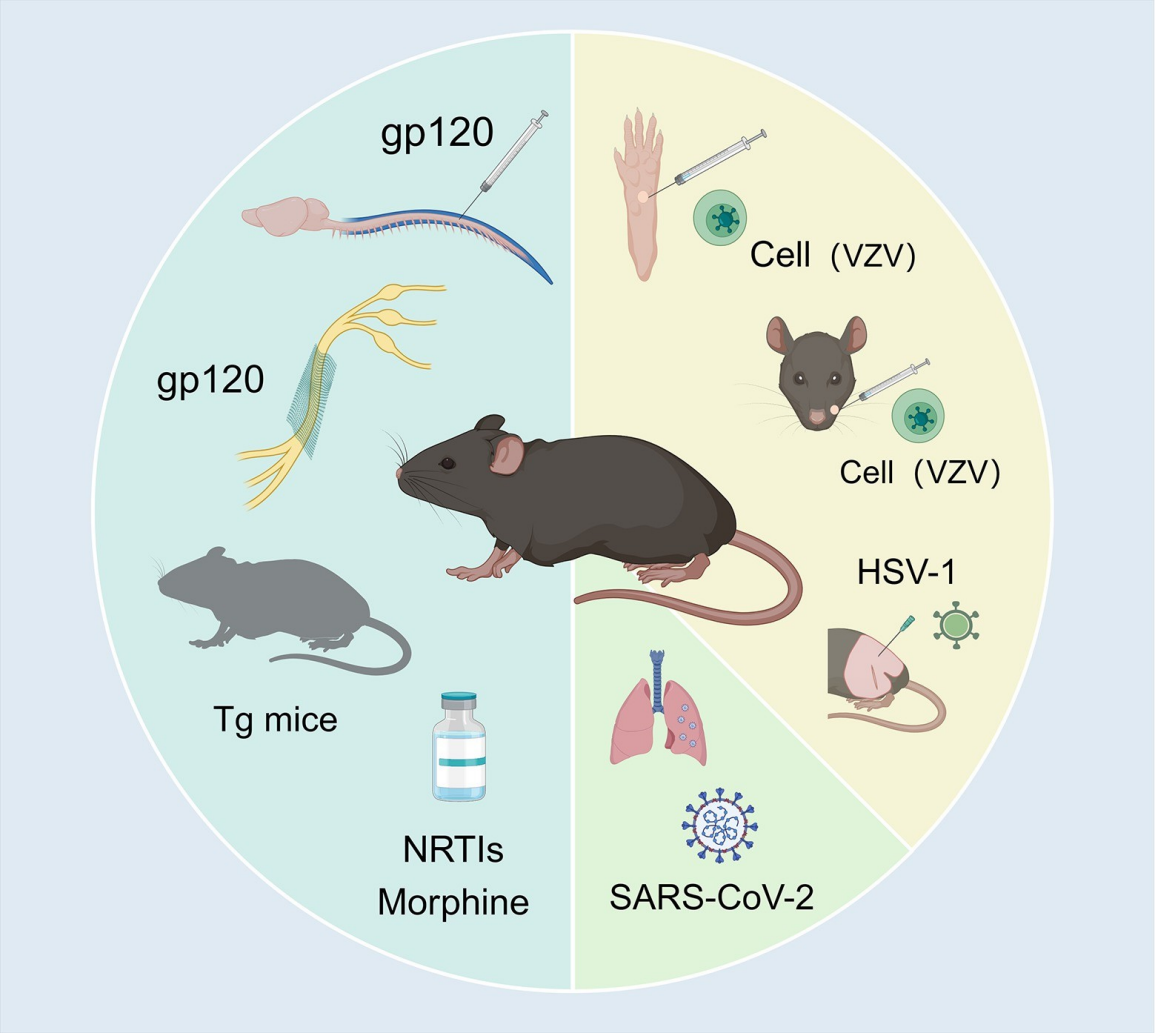
Various virus-induced neuropathic pain model. (Blue part) The HNP model can be established by intrathecal injection of gp120. Mice are administered gp120 intrathecally (5 μl, 20 ng/μl) on days 0, 3, and 7 [38]. One to 2 h postinjection, the mechanical withdrawal threshold and thermal withdrawal latency are significantly reduced, and the symptoms can persist for 21 days. This mouse model shares many similarities in pathological manifestations with pain in HIV-1–positive patients, such as pain behaviors, peripheral nerve lesions, activation of glial cells, synaptic degeneration, and abnormal activation of spinal dorsal horn pain-related signaling pathways. And the peripheral injury model can also be established by loosely wrapping oxidized regenerated cellulose around the sciatic nerve and soaking it in physiological saline containing 200 ng of gp120 [9]. Mechanical and thermal hyperalgesia can persist for more than 2 weeks. Conditional HIV-1 Tat transgenic mice, given doxycycline at 100 mg/kg per day intraperitoneally for 14 days, can conditionally induce Tat protein expression in the central nervous system to establish a model [8]. The HIV-1 Tg model can mimic defects observed in patients, such as changes in the nervous and immune systems, while also observing sensitization of pain-related behaviors [39]. Some antiretroviral drugs, especially those with higher mitochondrial toxicity, may affect the normal function of nerve cells. Continuous intraperitoneal injections of dideoxycytidine (ddc) (25 mg/kg) in mice for 5 days can induce mechanical stimulus sensitization, with no difference observed in sensitivity to heat or cold stimuli [40]. In HNP model, gp120 was intrathecally injected into mice on days 0, 3, 5, 11, and 16. Morphine was repeatedly injected into gp120 mice at a dose equivalent to the high end of clinical application (intraperitoneal, 20 mg/kg) on the same day [41]. (Yellow part) Since VZV cannot directly infect rodents, researchers have established models by inoculating African green monkey kidney fibroblast solutions infected with VZV or clinical patients’ herpes content into the toes or subcutaneous tissue of rats, leading to the onset of mechanical and thermal hypersensitivity after a certain period of infection [42]. Similarly, symptoms of trigeminal zoster can be simulated by injecting VZV-infected cells into the whisker pads [43]. Percutaneous inoculation of HSV-1 can induces herpes zoster-like skin lesions in mice [44]. When HSV-1 is locally inoculated into the hind limbs (tibia or femur), a small number of vesicles appear on the dorsal surface of the animals on the fifth day postinoculation, followed by herpes zoster-like skin lesions on days 6–10 postinoculation, which almost disappear by day 20. (Yellow part) Researchers inoculated 2–3-month-old hamsters with 100 μl of PBS containing 1,000 plaque-forming units (PFUs) of SARS-CoV-2 and 100,000 PFUs of IAV via intranasal route [34]. Within the first 24 h of nasal viral infection, SARS-CoV-2 transcripts were detected in the cervical, thoracic spinal cord, and DRGs. (Green part) Additionally, SARS-CoV-2–infected hamsters exhibited mechanical allodynia for approximately 1 month. Moreover, transcriptional characteristics closely resembled those of models of persistent inflammation and nerve damage [45]. Figure was produced using BioRender (IB26ZAQP1X).

Viral infections can lead to neuropathic pain through various mechanisms, resulting in different models for studying this phenomenon. Here, we explore the neuropathic pain models associated with HIV [8,9,38–41], VZV [42–44], and SARS-CoV-2 [34,45], highlighting their unique characteristics and the insights they offer into the pathophysiology of virus-induced neuropathic pain (**Fig 1**).

## Latent molecular mechanisms of HIV-NP

**Fig 2:**
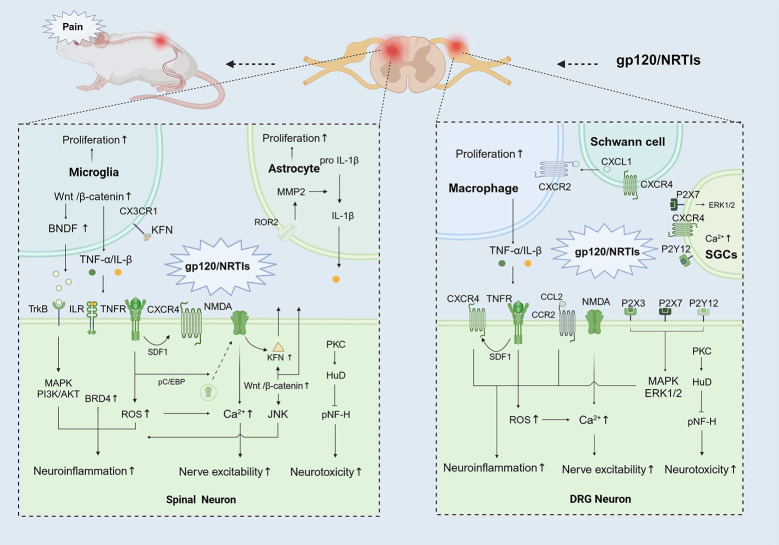
Potential molecular mechanisms in HNP models. Under the stimulation of gp120/NRTIs, the expression of various molecules/receptors changes, leading to neuroinflammation, glial cell proliferation, and neuronal damage, ultimately resulting in neuropathic pain. (A) In spinal cord. The expression levels of Wnt3a and β-catenin increased in microglia, thereby regulating the expression of BDNF and impacting hypersensitivity. HIV-1 gp120 induces synaptic degeneration in the spinal pain neural circuit by activating microglia via Wnt3a/β-catenin–regulated FKN expression in neurons. Gp120 also regulates astrogliosis, which promotes the expression of hyperalgesia and neuropathic pain through a Wnt5a-ROR2-MMP2 axis. Administration of ddC up-regulates NF-M and pNF-H proteins, while HuD competitively inhibits the increase in pNF-H levels by binding to BDNF mRNA. (B) In DRG. Gp120 increased P2X7 expression, IL-1β and TNF-α levels, ERK1/2 phosphorylation levels, and also enhanced IL-10 expression in the SGCs and neuron. Schwann cell-derived CXCL1, secreted in response to gp120 exposure, is responsible for macrophage infiltration. In the DRG and spinal dorsal horn, administration of gp120 induces up-regulation of TNF-α, CXCR4, and SDF1α. TNF can regulate CXCR4 expression through SDF1. HIV gp120 induces glial cell activation, leading to the release of TNF-α. TNF binds to its receptor TNFR on neurons, causing an increase in mitochondrial ROS, which can activate various other cellular signaling pathways. Mitochondria also participate in the regulation of cellular Ca2+ homeostasis. BNDF, brain-derived neurotrophic factor; MMP2, matrix metalloproteinase 2; ROR2, receptor tyrosine kinase-like orphan receptor 2; NRTIs, nucleoside reverse transcriptase inhibitors; CX3CR1, CX3C chemokine receptor 1; TNFR, tumor necrosis factor receptor; CXCR4, C-X-C chemokine receptor type 4; NMDA, N-Methyl-D-Aspartate; BRD4, bromodomain-containing protein 4; SDF1, stromal-derived factor 1; Ca2+, calcium ions; ROS, reactive oxygen species; JNK, c-Jun N-terminal kinase; PKC, protein kinase C; HuD, human antigen D; NF-H, neurofilament heavy chain. Figure was produced using BioRender (IB26ZAQP1X).

HIV specifically infects cells of the human immune system, and therefore, under natural conditions, it typically does not infect animals. In basic research, administration of HIV gp120 protein to animals can simulate the direct impact of HIV infection on the nervous system [46]. HIV transgenic animals or nucleoside reverse transcriptase inhibitors (NRTIs) can also induce related neuropathic changes and sensory abnormalities, greatly advancing research on HIV-related pain (**Fig 1, blue part**). Next, we will outline the common characteristics of HIV-associated neuropathic pain models and the current molecular mechanisms **(Fig 2 and S2 Table)**.

### HIV protein-induced HNP models

Gp120, Tat, and Vpr are key toxic proteins of HIV [19]. Infected microglial cells and astrocytes can release gp120 into the local microenvironment, which exhibits neurotoxicity and has been found to induce axonal degeneration, dendritic damage, and synaptic loss in primary neurons [47]. Additionally, gp120 can be recognized by CD4 receptors on target cell membranes [48], thereby promoting further activation and proliferation of neuroimmune cells [49]. Both intrathecal and peripheral administration of gp120 protein can simulate symptoms of human HNP [47,50]. Yuan and colleagues found that Wnt5a regulates the pathogenesis of gp120-induced pain by sensitizing pain-processing SDH neurons through the JNK/TNF-α signaling pathway [51]. Ru and colleagues discovered that HIV-1 gp120 induces synaptic degeneration in the spinal pain neural circuit by activating microglia via Wnt3a/β-catenin–regulated FKN expression in neurons [52]. Interestingly, the Wnt3a/β-catenin pathway is up-regulated in microglial cells, leading to overexpression of BDNF, which can be recognized by TrkB receptors on the surface of neurons, thereby causing neuroinflammation [53]. HIV gp120 induced overexpression of TNFR. It increases mitochondrial ROS and leads to the activation of the transcription factor pCREB, which promotes the recycling of NMDA receptors from endosomes to the plasma membrane [22]. Interleukin receptors also appear to play an important role in this process. In the study by Skinner and colleagues, IL-6 may exert its effects on spinal cord glial cells and/or neurons, potentially through autocrine or paracrine pathways, to promote gp120-induced heightened pain sensitivity [54].

Furthermore, local administration of gp120 can establish a model of peripheral nerve injury. Studies have found a close association between P2Y12 and gp120-induced neuropathic pain. Treatment of DRG Satellite Glial Cells with P2Y12 short hairpin RNA (shRNA) reduced the up-regulation of P2Y12 mRNA and protein expression in DRG SGCs, thereby alleviating mechanical and thermal hyperalgesia induced by gp120 treatment in rats [55]. GABA receptor activity appears to be closely related to this model, as peripheral gp120 induced a reduction in GABA immunoreactivity, an increase in signals of mitochondrial superoxide, and up-regulated immunoreactivity expression of pCREB and pC/EBPβ in the spinal dorsal horn [56]. The interaction between peripheral immune cells is also noteworthy. Schwann cell-derived CXCL1, secreted in response to gp120 exposure, is responsible for macrophage infiltration into peripheral nerves, thereby associated with pain-like behaviors in mice [57].

Despite intervention with antiretroviral drugs, it continues to be expressed in host cells and persists in central nervous system tissues, leading to neuroinflammation and subsequent neurotoxicity [58]. The Tat protein primarily participates in neuropathic pain by activating microglia and inducing oxidative stress [59]. Tat is involved in reducing nerve fiber density in both male and female mice, increasing the average amplitude of sensory nerve action potentials, inducing pain-related behaviors, and down-regulating the expression of the PPAR-α gene in the spinal cord [60]. After Tat-induced microglial activation, excessive production of pro-inflammatory cytokines is promoted through the activation of the p38MAPK and NF-kB signaling pathways [61]. Additionally, Tat may mediate toll-like receptor-dependent neuropathic pain behaviors [62]. The Vpr appears early after HIV infection and is a highly conserved gene that plays a crucial role in virus infection, replication, and spread, making it an intriguing therapeutic target [63]. Elevated levels of Vpr can be detected in the blood and cerebrospinal fluid in the late stages of the disease. As an extracellular protein, Vpr triggers apoptotic pathways, stimulates the release of inflammatory factors, interferes with ATP production, and leads to the accumulation of reactive oxygen species and increased oxidative stress [64]. Similar to gp120, the role of Vpr in central nervous system symptoms and HIV-related neurocognitive changes is a common focus, and its potential neurotoxicity in the context of neuropathic pain remains to be explored.

### Transgenic HNP models

The HIV-1 transgenic (HIV-1 Tg) model shares many similarities with HIV-1–infected individuals in terms of immunoreactivity and pathology [39]. Therefore, HIV-1 Tg mice serve as an invaluable model for investigating the pathogenesis of chronic HIV-1–related conditions, providing a safe, reproducible, and cost-effective methodology. Since rodents generally cannot express the infectious HIV-1 virus, transgenic mice are engineered to express specific HIV-1 proteins, such as the envelope protein gp120 and the regulatory protein Tat [65]. In a study, induction of Tat expression produced abnormal pain responses to mechanical or cold (but not heat) stimuli, lasting at least 2 to 3 weeks for mechanical hypersensitivity and at least 8 weeks for cold hypersensitivity [60]. Therefore, it can serve as a neuropathic model to investigate pain-related mechanisms and treatment approaches. Studies have found that HIV-1 Tg rats exhibit similar behavior to those with HIV-associated neuropathy, specifically cold sensitivity, associated with dynamic changes in oxidative stress, expression of gliotic markers, and integrity of the BBB [8]. Acharjee and colleagues found that Vpr caused DRG neuronal damage, likely through cytosolic calcium activation and cytokine perturbation [66]. The TrkA receptor agonist indicated that nerve growth factor acted through the TrkA to counteract the Vpr-mediated decrease in axon outgrowth in DRG [67].

### Antiviral drug-induced HNP models

NRTIs can place HIV patients in a state of extremely low viremia, significantly reducing the mortality rate associated with HIV infection. Similar to neuropathic pain induced by chemotherapy drugs, prolonged use of some NRTIs may exert toxic effects on the nervous system, leading to neuroinflammation or neuropathy, thereby causing pain [68]. Interestingly, Munawar and colleagues found that administration of antiretroviral drugs induced thermal hyperalgesia in mice [69]. With the use of these drugs, pathological changes in nerve tissues can be observed, such as neuronal degeneration, axonal injury, and activation of glial cells. Sanna and colleagues found that JNK3 plays a critical role in regulating ddC neurotoxicity-induced mechanical pain hypersensitivity, while JNK1 is important for the activation of c-Jun and GAP-43 as part of an essential pathway in a regeneration program [70]. The presence of a HuD–BDNF–NF-H pathway is activated as a regenerative response to axonal damage induced by ddC treatment, countering the antiretroviral neurotoxicity [71]. Similarly, ddC induced notable neuroinflammation in the spinal cord, as evidenced by the up-regulation of pro-inflammatory cytokines TNF-α and IL-1β, along with microglial and astrocytic responses mediated by Wnt5a signaling [72].

### Opioid drug-induced HNP models

Opioids alleviate pain by acting on opioid receptors in the central nervous system [73]. However, long-term use or abuse of opioids may lead to some side effects, including hyperalgesia and increased sensitivity to non-painful stimuli [3,73–75]. When neuropathic pain in HIV patients is difficult to control with conventional medications, opioids can alleviate pain to some extent, but prolonged and excessive use of opioids may increase the risk of HNP. For one thing, prolonged and excessive use of opioids may damage the nervous system, thereby increasing the occurrence and severity of neuropathic pain [76]. Additionally, opioids may suppress the immune system, reduce the activity of immune cells, thereby increasing the risk of HIV virus replication and disease progression, indirectly increasing the occurrence [77]. The study found that morphine exacerbates the development of HIV-associated pain, including astroglia activation, pro-inflammatory cytokine expression, and Wnt5a signaling [41]. Gp120 diminishes morphine antinociception in the PAG, but this effect can be restored by blocking CXCR4 receptors [78]. Moreover, gp120/M increased the expression of spinal TNFRI, mitochondrial superoxide, and cleaved caspase-11 [79].

## Potential molecular mechanisms of *VZV-NP*

**Fig 3:**
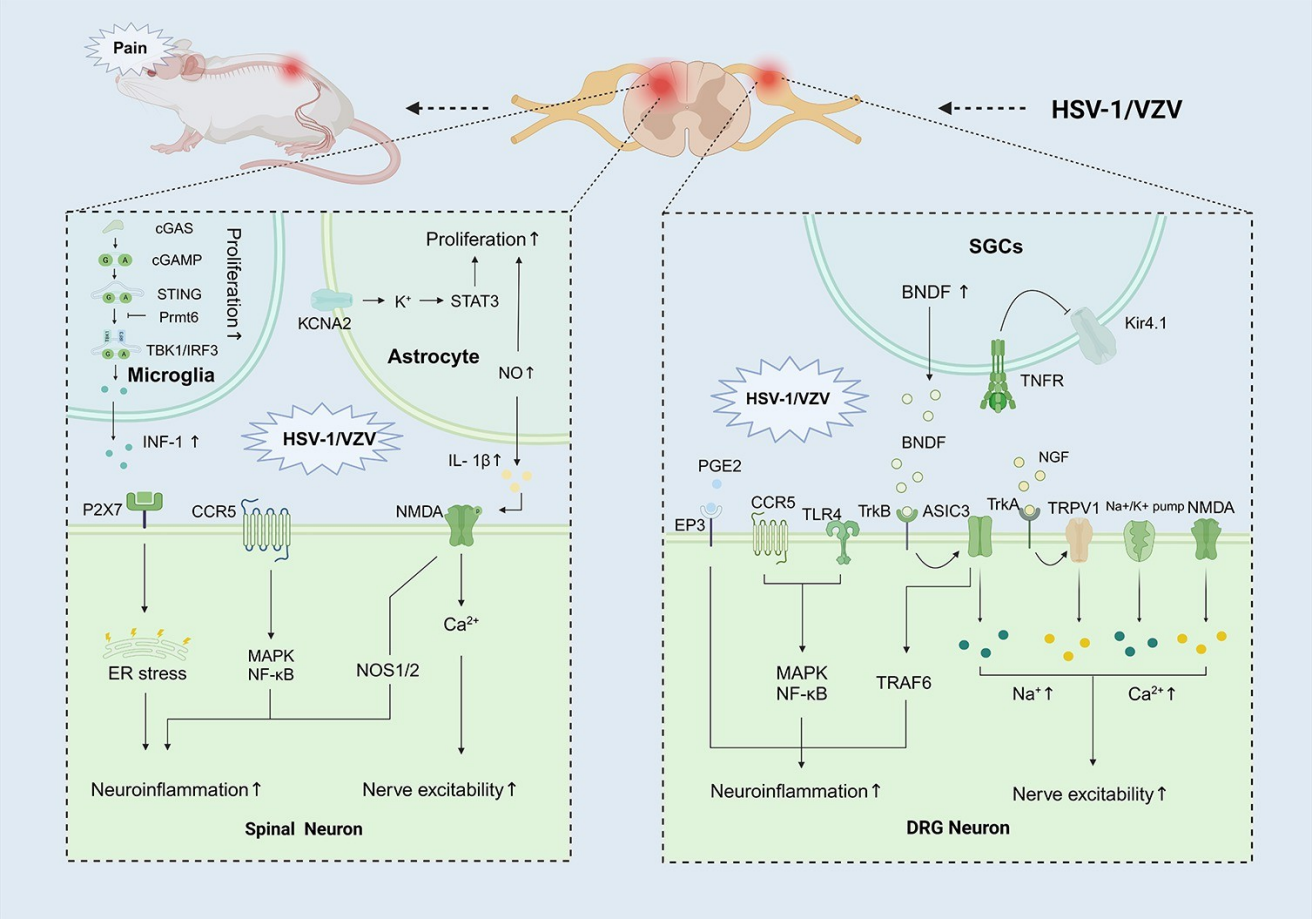
Potential molecular mechanisms in PHN models. Upon stimulation by HSV-1/VZV infected cells, alterations occur in the expression of various molecules/receptors, culminating in neuroinflammation, glial cell proliferation, and neuronal damage, ultimately giving rise to neuropathic pain. (A) In spinal cord. HSV-1 is rapidly recognized by astrocytic cGAS, leading to self-activation of cGAS, thereby inducing the synthesis of cGAMP. Subsequently, cGAMP binds to STING, resulting in the phosphorylation of TBK1, which in turn stimulates the phosphorylation and nuclear translocation of IRF3, thereby promoting the transcription and production of IFN-I. Prmt6 mediates STING inactivation through methylation, reducing the phosphorylation of TBK1 and IRF3, resulting in the inhibition of IFN-I production and antiviral innate immunity. KCNA2-AS participates in PHN partly by enhancing the translocation of pSTAT3 from the cytoplasm to the nucleus and promoting the activation of spinal astrocytes. The P2X7 receptor antagonist BBG mitigates PHN by triggering ER stress activation and diminishing pyroptosis. NOS2 and NOS1 are responsible for herpetic and postherpetic allodynia. (B) In DRG. The production of TNF, mediated by TNF/TNFR1 signaling in SGCs, down-regulates the expression of potassium channels, indirectly enhancing the excitability of primary sensory neurons and ultimately leading to the development of herpetic neuralgia. Similarly, in PHN models, alterations in the expression of receptors such as TRPV1, sodium ion channels, calcium ion channels, and TLR4 can induce changes in the excitability of DRG neurons, leading to persistent pain. cGAS, cyclic GMP-AMP synthase; cGAMP, cyclic GMP-AMP; STING, stimulator of interferon genes; Prmt6, protein arginine methyltransferase 6; TBK1, TANK-binding kinase 1; IRF3, interferon regulatory factor 3; STAT3, signal transducer and activator of transcription 3; KCNA2, potassium voltage-gated channel subfamily A member 2; P2X7, P2X purinoceptor 7; CCR5, C-C chemokine receptor type 5; CCL5, C-C motif chemokine ligand 5; NMDA: N-Methyl-D-Aspartate; NOS1/2, nitric oxide synthase 1/2; SGCs, satellite glial cells; TNFR, tumor necrosis factor receptor; PAQR, progestin and AdipoQ receptor; TrKA, tropomyosin receptor kinase A; TRPV1, transient receptor potential vanilloid 1; Na+, sodium ion; Ca2+, calcium ion; TLR4, toll-like receptor 4; PI3K, phosphoinositide 3-kinase; AKT, protein kinase B. Figure was produced using BioRender (PK26ZAQEIO).

Human is the only natural host of the VZV virus, and latent herpes zoster virus cannot be activated in animals [80] (**Fig 1, yellow part**). Thus, it is of paramount importance to search for preclinical models with clinical symptoms similar to PHN, as it can help us better understand the pathophysiological processes, develop new therapeutic approaches, and provide more effective treatment strategies for clinical practice **(Fig 3)**.

### Plantar/Whisker pad VZV injection model

In the VZV model, NO triggers spinal astrocytic activation. Activated astrocytes then up-regulate IL-1β expression, leading to NMDAR phosphorylation in spinal dorsal horn neurons, intensifying pain transmission [81]. Garry and colleagues found that VZV infection induced an increased behavioral reflex responsiveness to both noxious thermal and mechanical stimuli ipsilateral to injection by spinal NMDA receptors, accompanied by up-regulation of sodium-calcium channels and ATF-3 expression [82]. In the whisker pad VZV injection model, it suggests a mechanism for pain induction involving the early expression of IE4 or IE63 proteins in abortively infected neurons after herpes zoster, potentially leading to aberrant host pain signaling and the development of PHN [43]. Although the model exhibits prolonged hyperalgesia and abnormal pain sensation, with behavioral manifestations highly resembling the occurrence, development, and resolution process of clinical PHN, it does not simulate the manifestations of chickenpox and acute pain, nor does it mimic the natural state of latency and reactivation.

### HSV-1 model

In this model, HSV-1 DNA replication and proliferation can be detected in the lumbar DRG of mice postinoculation [83]. Mice exhibit abnormal mechanical allodynia relatively early, usually coinciding with the appearance of vesicles, while sensitization to temperature stimuli occurs later in the course of herpes zoster. But this model is usually associated with the acute phase of primary or recurrent infection, and sensory abnormalities typically decrease gradually with the disappearance of the vesicles, with a shorter duration. In contrast, PHN is a complication of herpes zoster infection that often occurs after the infection has resolved, with persistent pain sensations even after the vesicles have disappeared. Kong and colleagues found that the propagation of HSV-1 in the DRG produces allodynia and hyperalgesia, escapes antiviral innate immunity, and results in PHN by up-regulating Prmt6 expression and inhibiting the cGAS-STING pathway [84]. Additionally, the inhibition of CCR5 demonstrated a significant analgesic effect and effectively alleviated the increase of inflammatory cytokines in both the DRG and spinal cord induced by HSV-1 infection in mice [85]. Furthermore, NOS2 and NOS1 may also be responsible for herpetic and postherpetic allodynia [86]. However, differences still exist between the model and human VZV infection.

## Underlying molecular mechanisms of SARS-Co-2—NP

In neurological research, there is currently a greater emphasis on studying conditions such as neuroinflammation and cognitive impairment following Coronavirus Disease 2019 (COVID-19), while investigations into the mechanisms of chronic neuropathic pain remain relatively scarce [87]. However, the manifestation of such sensory abnormalities is noteworthy. Interestingly, HIV can induce primary sensory neuropathy by interacting with viral proteins and axons, simultaneously inducing secondary inflammation at these nerve sites, thus leading to symptoms of hyperexcitability [62]. Herpes zoster virus latent in DRGs exhibits neurotropism and directly induces abnormal activity in these primary sensory cells upon reactivation [82]. However, the mechanism underlying sensory abnormalities induced by coronaviruses remains unclear. Consequently, experiments utilizing SARS-CoV-2–infected hamsters have been conducted to study the impact on somatosensory symptoms [45] (**Fig 1, green part**). SARS-CoV-2–infected hamsters exhibited mechanical allodynia for approximately 1 month [45]. Moreover, transcriptional characteristics closely resembled those of models of persistent inflammation and nerve damage. This advances the understanding of sensory abnormalities associated with SARS-CoV-2. However, directly utilizing the virus for basic experiments in conventional laboratories is often impractical due to the high laboratory requirements, limiting direct research into the virus’s effects on nerves. Researchers have thus employed pathogenic viral proteins to simulate infection states and explore their molecular mechanisms, focusing on proteins such as the E protein and S protein [88].

The E protein of SARS-CoV-2 induces neuroinflammation via TLR2, leading to depression-like behavior and olfactory impairment in mice [89]. Similarly, the S1 protein can activate BV-2 microglial cells, resulting in the production of pro-inflammatory mediators. Experimental findings suggest that the induction of neuroinflammation by this protein in microglial cells is mediated through the activation of NF-κB and p38 MAPK, closely associated with TLR4 activation [90]. Intrathecal injection of pathogenic proteins to induce sensory abnormalities in rodents simulates a model of chronic neuropathic pain, aiming to explore potential mechanisms beyond the interaction between the virus and neuroglial cells neurons [90]. Furthermore, attention should be paid to factors influencing chronic pain resulting from the virus’s effects on peripheral nerve sites. These studies contribute to a deeper understanding of the mechanisms by which the novel coronavirus affects the nervous system, thereby providing new insights and approaches for the treatment and management of neuropathic pain.

## Sex differences

Neuropathic pain induced by HIV infection exhibits certain gender differences. Guindon and colleagues found in their study on gp120-induced neuropathic pain that compared to male mice, female mice showed increased mechanical allodynia and cold sensitivity [38]. Similarly, in mouse neuropathic pain induced by antiretroviral drugs, changes in spinal cord neuroimmune cells and molecules were gender dependent, with female mice being more prone to neuroimmune alterations than male mice [91]. Female mice exhibited a more significant impact on neuropathic hypersensitivity, while male mice showed a more pronounced effect on inflammatory hypersensitivity.

Another study involving transgenic expression of HIV-1 Tat induced by doxycycline in male and female mice found that compared to males, females experienced greater magnitudes of mechanical pain and abnormal cold sensation, with mechanical allodynia gradually increasing over time. Acute morphine or gabapentin treatment partially alleviated pain abnormalities in males but showed no significant effects in females [60]. Similarly, PHN also exhibits certain gender differences. Kramer and colleagues found that during the development of herpes zoster-related pain, Neurexin 3α, closely associated with GABA release, seemed to play a more important role in pain development in female rats compared to males [92]. Clinical studies also support this finding. Tsao and colleagues investigated gender differences in pain and prescription opioid misuse among HIV-infected individuals. They found significant gender disparities, with women being more likely to report pain and engage in prescription opioid misuse compared to men [93]. Conversely, men reported less pain and were less likely to misuse prescription opioids.

In a clinical study on the development of PHN, age and gender were found to have a close interaction. Specifically, younger women and older men were more likely to develop PHN, while younger men and older women were relatively less symptomatic of PHN [94]. This indicates the important roles of gender and age factors in the development of PHN, with gender differences possibly being modulated by age factors. These data reveal gender differences in virus-related neuropathic pain. In light of these gender differences, it is recommended to focus on gender factors in the treatment of neuropathic pain and consider the pain perception and treatment needs of different patients comprehensively. This may require personalized treatment strategies, including gender-specific pharmacological treatment plans, to more effectively alleviate pain symptoms. Furthermore, a deeper understanding of gender differences can help uncover gender-related factors in the pathogenesis of neuropathic pain, providing more precise guidance for future pain management.

## Conclusion and future perspectives

In this review, we have described the potential molecular mechanisms of various neuropathic pain models, highlighting the diverse impacts of viruses on the nervous system. Understanding these mechanisms across different viral infections contributes significantly to our comprehension of neuropathic pain pathophysiology.

These viruses exhibit similarities and differences in their effects on the nervous system. In terms of similarities, the neurological inflammation and damage triggered by these viral infections may result from direct viral invasion of neurons and surrounding tissues, or from excessive activation of the immune system and release of inflammatory mediators [72]. Firstly, they can all enter the nervous system through the bloodstream and infect neurons through retrograde transmission along nerves. These viruses may replicate within the nervous system, leading to inflammation and neuronal damage [54]. And specific pathways associated with neuropathic pain include the abnormal activity of sodium channels, calcium channels, and NMDA receptors [22]. These inflammatory mediators may directly damage neurons and surrounding nerve tissues or lead to neuronal hyperexcitability and conduction abnormalities, thereby causing neuropathic pain.

However, key differences still exist among these viruses: (1) Virus specificity: HIV is a retrovirus targeting immune cells; VZV is a double-stranded DNA virus affecting the skin and nervous system; SARS-CoV-2 is a single-stranded RNA virus primarily entering through the respiratory tract, impacting multiple systems. (2) Infection sites and extent: HIV typically causes lesions in the spinal cord’s white and gray matter [20]; VZV targets sensory ganglia and cutaneous nerves [24]; SARS-CoV-2 can cause widespread nervous system damage. These differences result in varying types and severities of neuropathic pain. (3) Onset and progression: HIV infection is chronic and progressive, with neurological complications developing over years; VZV infection can lead to acute PHN lasting months to years [25]; SARS-CoV-2–related neuropathic pain may arise during acute infection or recovery, with variable symptom durations. (4) Treatment strategies: HIV-related neurological complications often require antiretroviral and immunomodulatory therapy; VZV-induced PHN needs antiviral and analgesic treatment; SARS-CoV-2–induced neuropathic pain may necessitate a comprehensive approach including symptomatic and immunomodulatory therapy.

Despite the advancements in animal and in vitro models, several challenges remain unresolved. Firstly, they fail to fully replicate the complexity and specificity of human virus infection, impeding an accurate reflection of infection’s impact on the nervous system. Discrepancies in disease progression and symptoms between animal models and humans restrict the precise simulation of neuropathic pain by the models. Secondly, accurately mimicking the influence of drug abuse on pain development in patients is another significant challenge. Current models may not entirely replicate the pathological changes in the nervous system caused by infection [95]. Additionally, existing models often struggle to accurately replicate the latency and reactivation phases of viruses within the nervous system. It is especially critical for understanding chronic neuropathic pain conditions, such as PHN, where the reactivation of latent viruses plays a pivotal role. Meanwhile, mechanisms are highly heterogeneous, involving multiple pathways and factors. This complexity makes it difficult to develop models that can accurately reflect the diverse pathophysiological processes. As previously mentioned, gender differences significantly influence the development and experience of neuropathic pain, yet current models often fail to adequately represent these differences. An ideal model of virus-associated neuropathic pain should successfully simulate viral infection and its impact on the nervous system, including neuronal damage and immune responses. Furthermore, it should accurately replicate the latency and reactivation processes of the virus within the nervous system, wherein viral gene replication is halted during latency but can be reactivated under stimulation to produce infectious viral particles. Behaviorally, the model should exhibit characteristic features associated with neuropathic pain, such as mechanical allodynia, thermal hyperalgesia, and tactile allodynia. Ultimately, the model should demonstrate stability, reproducibility, and offer valuable insights for the development of clinical treatment strategies.

Similarly, the therapeutic management of virus-associated neuropathic pain faces numerous challenges. While pregabalin or gabapentin are first-line treatments for HNP and PHN, their efficacy is limited. A significant portion of patients report only partial relief from pain symptoms. Both pregabalin and gabapentin are associated with side effects such as dizziness, sedation, and peripheral edema, which can limit their tolerability and long-term use [10,11]. There is considerable variability in patient response to these medications, with some patients experiencing minimal or no benefit. Currently, analgesic strategies include the use of pregabalin combined with spinal cord stimulation or opioids, neuromodulation [12]. For HNP, antiretroviral drugs also face challenges in treating central nervous system infections due to poor BBB penetration. In recent years, significant progress has been made in overcoming the barriers with drug delivery systems. Key technologies include carrier-mediated transport systems [96], such as proteins or peptides as carriers, enhancing drug penetration by binding to specific transporters on the BBB. Additionally, nanotechnology applications are widespread [97], utilizing carriers like polymer nanoparticles, liposomes, or microbubbles to effectively facilitate drug passage across the BBB and release into target areas.

Furthermore, substantial progress is anticipated in in vitro experimental investigations of virus-induced neuropathic pain with the integration of contemporary cell reprogramming methodologies [98]. Leveraging the potential of reprogramming, specialized neuronal cell types pertinent to neuropathic pain pathways could conceivably be generated. These engineered neuronal models hold considerable promise as indispensable instruments for elucidating the intricate mechanisms underlying viral neuropathogenesis and ensuing pain states. Furthermore, coupling cell reprogramming with sophisticated culture systems [99], such as organoids or multicellular tissue models, has the potential to augment the physiological significance and intricacy of these in vitro platforms. Such amalgamation might facilitate the recapitulation of pivotal aspects of the neural microenvironment impacted by viral infections, thereby enabling more faithful modeling of neuropathic pain conditions. Additionally, the employment of single-cell transcriptomics and other omics methodologies in conjunction with reprogrammed cell models could provide deeper insights into the molecular signatures. In summary, by capitalizing on the capabilities of cell reprogramming technologies, we are poised to advance our comprehension of virus-induced neuropathic pain and accelerate the development of innovative therapeutic strategies to alleviate this incapacitating condition.

In summary, future research will delve more deeply into the pathological mechanisms of virus-related neuropathic pain, accelerate the discovery and clinical translation of new drugs, and provide more innovative and effective solutions to address this clinical challenge, ultimately improving treatment outcomes and quality of life for patients.

## Supporting information

S1 Table Neurobiological characteristics of different viruses. We compared the biological characteristics of the 3 viruses. HIV, human immunodeficiency virus; VZV, varicella zoster virus; SARS-CoV-2, Severe Acute Respiratory Syndrome Coronavirus 2; gp120, Glycoprotein 120; gp41, Glycoprotein 41; CD4, cluster of differentiation 4; S, spike protein; ACE2, angiotensin-converting enzyme 2; CNS, central nervous system; DRG, dorsal root ganglia.
(DOCX)

S2 Table Potential molecular mechanisms in different HNP models. The table presents potential mechanisms of different HNP models, including information on model types, potential pathways, biofunctions, and other relevant details. “/” means not mentioned in the article. BDNF, brain-derived neurotrophic factor; ICR mice, Institute of Cancer Research mice; JNK, c-Jun N-terminal kinase; TNF-a, tumor necrosis factor-alpha; SDH, succinate dehydrogenase; P2X7, purinergic receptor P2X, ligand-gated ion channel 7; ERK1/2, extracellular signal-regulated kinase 1/2; FKN, fractalkine; CX3R1, CX3C chemokine receptor 1; NF-κB, nuclear factor kappa B; CSF, cerebrospinal fluid; Ca2+, calcium ion; AchRs, acetylcholine receptors; CREB, cAMP response element-binding protein; ROR2, receptor tyrosine kinase-like orphan receptor 2; MMP2, matrix metalloproteinase-2; IL, interleukin; GFAP, glial fibrillary acidic protein; P2Y12, purinergic receptor P2Y, G-Protein coupled 12; Akt, protein kinase B; DRG, dorsal root ganglion; P2X3, purinergic receptor P2X, ligand-gated ion channel 3; CXCR4, C-X-C chemokine receptor type 4; SDF1, stromal cell-derived factor 1; GABA, gamma-aminobutyric acid; CXCL1, C-X-C motif chemokine ligand 1; CCR2, C-C chemokine receptor type 2; CCL2, C-C motif chemokine ligand 2; CGRP, calcitonin gene-related peptide; IB4, isolectin B4; NRTI, nucleoside reverse transcriptase inhibitor; 5-HT2A, 5-Hydroxytryptamine receptor 2A; MAPK, mitogen-activated protein kinase; CB, cannabinoid; GMP, guanosine monophosphate; PKG, protein kinase G; AMPK, AMP-activated protein kinase; AT2R, angiotensin II type 2 receptor; TRPV1, transient receptor potential vanilloid 1; TRPA1, transient receptor potential ankyrin 1; TrkA: tropomyosin receptor kinase A; PPARs, peroxisome proliferator-activated receptors; Brd4, bromodomain-containing protein 4.
(DOCX)

S3 Table Potential molecular mechanisms in different PHN models. The table presents potential mechanisms of PHN models, including information on model types, potential pathways, biofunctions, and other relevant details. “/” means not mentioned in the article. CCL5, C-C motif chemokine ligand 5; CCR5, C-C chemokine receptor type 5; HSV-1, herpes simplex virus type 1; DRG, dorsal root ganglion; TLR4, toll-like receptor 4; TNF, tumor necrosis factor; PHP, pseudohypoparathyroidism; K+, potassium ion; COX, cyclooxygenase; PGE2, prostaglandin E2; EP3, prostaglandin E2 receptor EP3; NMDA, N-Methyl-D-Aspartate; NR2Bs, N-Methyl-D-Aspartate receptor subunit 2B; NOS, nitric oxide synthase; Prmt6, protein arginine methyltransferase 6; cGAS, cyclic GMP-AMP synthase; STING, stimulator of interferon genes; VPM, ventral posterior medial nucleus; VPL, ventral posterior lateral nucleus; P2X7, purinergic receptor P2X, ligand-gated ion channel 7; BBG, brilliant blue G; ER stress, endoplasmic reticulum stress; KCNA2, potassium voltage-gated channel subfamily A member 2; STAT3, signal transducer and activator of transcription 3; pSTAT3, phosphorylated signal transducer and activator of transcription 3; MRC-5, medical research council cell strain 5; TRPV1, transient receptor potential vanilloid 1; NO, nitric oxide; vHPPE, ventriculoperitoneal hydrocephalus; VZV, varicella zoster virus; SNT, syntrophin; H3, histone H3; PWL, paw withdrawal latency; MWT, mechanical withdrawal threshold; TRX, thioredoxin; NP, nucleoside phosphorylase; DNF, brain-derived neurotrophic factor; ASIC3, acid-sensing ion channel 3; PAQR, progestin and AdipoQ receptor; VGAT, vesicular GABA transporter; ERK, extracellular signal-regulated kinase; IE4, immediate early protein 4.
(DOCX)
