# Supplementary material for: Exploring viral neuropathic pain: Molecular mechanisms and therapeutic implications
Source: PLoS Pathog. 2024 Aug 8;20(8):e1012397. doi: 10.1371/journal.ppat.1012397 (PMC11309435; doi:10.1371/journal.ppat.1012397)
Supplement: S1 Table — We compared the biological characteristics of the 3 viruses. HIV, human immunodeficiency virus; VZV, varicella zoster virus; SARS-CoV-2, Severe Acute Respiratory Syndrome Coronavirus 2; gp120, Glycoprotein 120; gp41, Glycoprotein 41; CD4, cluster of differentiation 4; S, spike protein; ACE2, angiotensin-converting enzyme 2; CNS, central nervous system; DRG, dorsal root ganglia. (DOCX) [file ppat.1012397.s001.docx]

**S1 Table. Neurobiological characteristics of different viruses**

| **Characteristics** | **HIV** | **VZV** | **SARS-CoV-2** |
| --- | --- | --- | --- |
| **Structure** | a single-stranded RNA virus, consisting of envelope, protein capsid, and reverse transcriptase. | a double-stranded DNA virus, containing envelope, protein capsid, and DNA genome. | a single-stranded RNA virus, characterized by helical symmetry envelope, protein capsid, and RNA genome. |
| **Pathogenic proteins** | It primarily utilizes two proteins, gp120 and gp41, to bind to the CD4 on host cells, facilitating entry and replication within the cells. | The main proteins include glycoproteins B, C, and E, which are involved in the entry and replication processes of the virus. | The primary pathogenic protein is the spike protein (S protein), which binds to the ACE2 receptor on host cells, mediating the entry of the virus into the cells. |
| **Initial Infection Route** | Blood, semen, vaginal secretions, breast milk | Respiratory droplets, direct contact | Respiratory droplets, potential olfactory nerve entry |
| **Primary Target Cells** | CD4+ T cells, monocytes, macrophages | Sensory neurons, satellite cells, dorsal root ganglia neurons | ACE2-expressing cells in respiratory epithelium, potential CNS |
| **Blood-Brain Barrier Crossing** | Yes | Yes | Yes |
| **Direct Nervous System Infection** | Yes, glial cells | Yes, neurons and glial cells | Yes, neurons and potentially glial cells |
| **Latency and Reactivation** | No direct latency, chronic inflammation in CNS | Yes, latent in dorsal root ganglia, reactivates as shingles | Possible latency and reactivation mechanisms |
| **Neurological Symptoms** | Peripheral neuropathy, cognitive impairment | Herpes zoster, post-herpetic neuralgia | Potential anosmia, neuroinflammatory symptoms |
| **Neuropathic Pain Characteristics** | Burning, tingling, numbness, cognitive dysfunction | Burning pain, allodynia, sensory loss | Muscle pain, potential neuropathic pain |
| **Inflammatory Response** | Chronic CNS inflammation, immune activation | Local and systemic inflammation | Neuroinflammation, cytokine storm |
| **Reference** | [1-5] | [6-9] | [10-14] |

We compared the biological characteristics of the three viruses. HIV: Human Immunodeficiency Virus VZV: Varicella-Zoster Virus; SARS-CoV-2: Severe Acute Respiratory Syndrome Coronavirus 2; gp120: Glycoprotein 120; gp41: Glycoprotein 41; CD4: Cluster of Differentiation 4; S: Spike Protein ACE2: Angiotensin-Converting Enzyme 2; CNS: Central Nervous System; DRG: Dorsal Root Ganglia

**Reference**

1. Sharp PM, Hahn BH. Origins of HIV and the AIDS pandemic. *Cold Spring Harbor Perspectives in Medicine*. 2011;1(1). doi:10.1101/cshperspect.a006841

2. Teixeira C, Gomes JRB, Gomes P, Maurel F. Viral surface glycoproteins, gp120 and gp41, as potential drug targets against HIV-1: Brief overview one quarter of a century past the approval of zidovudine, the first anti-retroviral drug. *European Journal of Medicinal Chemistry*. 2011;46(4):979-992. doi:10.1016/j.ejmech.2011.01.046

3. Annunziata P. Blood-brain barrier changes during invasion of the central nervous system by HIV-1: Old and new insights into the mechanism. *Journal of Neurology*. 2003;250(8):901-906. doi:10.1007/s00415-003-1159-0

4. Bowers K, Pitcher C, Marsh M. CD4: A Co-receptor in the immune response and HIV infection. *International Journal of Biochemistry and Cell Biology*. 1997;29(6):871-875. doi:10.1016/S1357-2725(96)00154-9

5. Lipton SA. HIV-Related Neurotoxicity. 99(1 991).

6. Kornfeind EM, Visalli RJ. Human herpesvirus portal proteins: Structure, function, and antiviral prospects. *Reviews in Medical Virology*. 2018;28(3):1-15. doi:10.1002/rmv.1972

7. Gershon AA, Breuer J, Cohen JI, et al. Varicella zoster virus infection. *Nature Reviews Disease Primers*. 2015;1(July):1-19. doi:10.1038/nrdp.2015.16

8. Reichelt M, Zerboni L, Arvin AM, Reichelt M, Zerboni L, Arvin AM. Mechanisms of Varicella-Zoster Virus Neuropathogenesis in Human Dorsal Root Ganglia Mechanisms of Varicella-Zoster Virus Neuropathogenesis in Human Dorsal Root Ganglia ᰔ. 2008;82(8). doi:10.1128/JVI.02592-07

9. The New England Journal of Medicine Downloaded from nejm.org at GAZI UNIVERSITESI MAIN LIBRARY on October 24, 2014. For personal use only. No other uses without permission. Copyright © 1990 Massachusetts Medical Society. All rights reserved. 1990.

10. Asselah T, Durantel D, Pasmant E, Lau G, Schinazi RF. COVID-19: Discovery, diagnostics and drug development. *Journal of Hepatology*. 2021;74(1):168-184. doi:10.1016/j.jhep.2020.09.031

11. Arya R, Kumari S, Pandey B, et al. Structural insights into SARS-CoV-2 proteins. *Journal of Molecular Biology*. 2021;433(2). doi:10.1016/j.jmb.2020.11.024

12. Barnes CO, Jette CA, Abernathy ME, et al. SARS-CoV-2 neutralizing antibody structures inform therapeutic strategies. *Nature*. 2020;588(7839):682-687. doi:10.1038/s41586-020-2852-1

13. de Melo GD, Perraud V, Alvarez F, et al. Neuroinvasion and anosmia are independent phenomena upon infection with SARS-CoV-2 and its variants. *Nature Communications*. 2023;14(1). doi:10.1038/s41467-023-40228-7

14. Khan M, Yoo SJ, Clijsters M, et al. Visualizing in deceased COVID-19 patients how SARS-CoV-2 attacks the respiratory and olfactory mucosae but spares the olfactory bulb. *Cell*. 2021;184(24):5932-5949.e15. doi:10.1016/j.cell.2021.10.027
