# Supplementary material for: Exploring viral neuropathic pain: Molecular mechanisms and therapeutic implications
Source: PLoS Pathog. 2024 Aug 8;20(8):e1012397. doi: 10.1371/journal.ppat.1012397 (PMC11309435; doi:10.1371/journal.ppat.1012397)
Supplement: S2 Table — The table presents potential mechanisms of different HNP models, including information on model types, potential pathways, biofunctions, and other relevant details. “/” means not mentioned in the article. BDNF, brain-derived neurotrophic factor; ICR mice, Institute of Cancer Research mice; JNK, c-Jun N-terminal kinase; TNF-a, tumor necrosis factor-alpha; SDH, succinate dehydrogenase; P2X7, purinergic receptor P2X, ligand-gated ion channel 7; ERK1/2, extracellular signal-regulated kinase 1/2; FKN, fractalkine; CX3R1, CX3C chemokine receptor 1; NF-κB, nuclear factor kappa B; CSF, cerebrospinal fluid; Ca2+, calcium ion; AchRs, acetylcholine receptors; CREB, cAMP response element-binding protein; ROR2, receptor tyrosine kinase-like orphan receptor 2; MMP2, matrix metalloproteinase-2; IL, interleukin; GFAP, glial fibrillary acidic protein; P2Y12, purinergic receptor P2Y, G-Protein coupled 12; Akt, protein kinase B; DRG, dorsal root ganglion; P2X3, purinergic receptor P2X, ligand-gated ion channel 3; CXCR4, C-X-C chemokine receptor type 4; SDF1, stromal cell-derived factor 1; GABA, gamma-aminobutyric acid; CXCL1, C-X-C motif chemokine ligand 1; CCR2, C-C chemokine receptor type 2; CCL2, C-C motif chemokine ligand 2; CGRP, calcitonin gene-related peptide; IB4, isolectin B4; NRTI, nucleoside reverse transcriptase inhibitor; 5-HT2A, 5-Hydroxytryptamine receptor 2A; MAPK, mitogen-activated protein kinase; CB, cannabinoid; GMP, guanosine monophosphate; PKG, protein kinase G; AMPK, AMP-activated protein kinase; AT2R, angiotensin II type 2 receptor; TRPV1, transient receptor potential vanilloid 1; TRPA1, transient receptor potential ankyrin 1; TrkA: tropomyosin receptor kinase A; PPARs, peroxisome proliferator-activated receptors; Brd4, bromodomain-containing protein 4. (DOCX) [file ppat.1012397.s002.docx]

**S2 Table. Potential Molecular Mechanisms in different HNP models**

| Model | Receptor/  pathway | Biofunctions | Cells | Tissue | Species | Ref. |
| --- | --- | --- | --- | --- | --- | --- |
| gp120-HNP | Wnt/β-catenin /BDNF | The expression levels of Wnt3a and β-catenin increased in the spinal cord of mice with herniated nucleus pulposus, thereby regulating the expression of BDNF and impacting hypersensitivity. | / | L4–L6 spinal cord | ICR mice | [1] |
| gp120-HNP | Wnt5a/JNK/TNF-a | Wnt5a regulates the pathogenesis of gp120-induced pain by sensitizing pain-processing SDH neurons through the JNK/TNF-α signaling pathway. | Neurons | L5-6 spinal cord | C57BL/6J mice | [2] |
| gp120-HNP | / | Gp120 play a critical role in the pathogenesis of HIV-associated pain. | U373-MAGI-CCR5 target cells | Plantar area of the hind paw；lumbar SDH of human and mouse | Female C57BL6 mice; Male SD rats | [3] |
| gp120-HNP | P2X7/ERK1/2 | Resveratrol alleviated mechanical hyperalgesia in gp120-treated rats by inhibiting the P2X7 receptor and reducing ERK1/2 phosphorylation levels. | HEK 293 cells | L4–6 DRGs | Male SD rats | [4] |
| gp120-HNP | / | Gp120-induced neuropathic pain-like behaviors exhibit sex-dependent. Female mice demonstrated heightened mechanical allodynia and increased sensitivity to cold compared to their male counterparts. | / | / | Female and male C57BL/6J mice | [5] |
| gp120-HNP | / | Oligodendrocytes exhibit specific reactivity within the SDH in both HIV-infected human patients and mice. | / | Lumbar SDHs | C57BL/6 mice and myelin-deficient mutant mice | [6] |
| gp120-HNP | / | Repeated exposure to gp120 also led to increased levels of neuronal RyR and mitochondrial superoxide within the spinal cord dorsal horn. | / | L4–5 spinal cord | Male SD rats | [7] |
| gp120-HNP | Wnt/β-catenin/ FKN/CX3R1 | HIV-1 gp120 induces synaptic degeneration in the spinal pain neural circuit by activating microglia via Wnt3a/β-catenin-regulated FKN expression in neurons. | Microglia; neurons | L4 –L5 SDH | Transgenic mice | [8] |
| gp120-HNP | / | IL-6 may exert its effects on spinal cord glial cells and/or neurons, potentially through autocrine or paracrine pathways, to promote gp120-induced heightened pain sensitivity. | / | The dorsal spinal cord | Male SD rats | [9] |
| gp120-HNP | / | The analgesic effect of buprenorphine is more effective than methadone in the HIV pain model. | / | / | Male SD rats | [10] |
| gp120-HNP | NF-κB | spinal cord NF-κB activation is involved in exaggerated pain states of gp120-HNP. | / | Spinal cord and CSF | Male SD rats | [11] |
| gp120-HNP | numerous | This study demonstrates significant differences in the molecular mechanisms of HNP during initiation, transition, and maintenance processes. | / | L3-L5 spinal cord | Male C57BL/6 mice | [12] |
| gp120-HNP | / | Minocycline can inhibit the activation of microglial cells and the expression levels of inflammatory factors induced by intrathecal HIV-1 gp120. | Microglia | Spinal cord and CSF | Male SD rats | [13] |
| gp120-HNP | endocannabinoid | In a rodent model of HIV-1-gp120 neuropathic pain, alterations in the gene expression of endocannabinoid (eCB) components occur in a sex-specific manner, with ovarian hormones playing a contributory role. | / | Spinal cord | Female and male C57BL/6 J mice | [14] |
| gp120-HNP | / | Gp120 evoked dose-dependent allodynia and induced a rapid elevation in intracellular free Ca2+ concentration within the dorsal horn cells of the spinal cord | / | Lumbar spinal cord | Male ddY mice and C57BL/6 mice | [15] |
| gp120-HNP | / | Activated glial cells in the dorsal spinal cord can exacerbate HIV-related pain states through the release of proinflammatory cytokines. | Microglia and astrocyte | Spinal cord and CSF | Male SD rats | [16] |
| gp120-HNP | AchRs | Choline significantly attenuated and reversed gp120-induced mechanical allodynia by suppressing microglial activation and the pro-inflammatory state. | Microglia | Lumbar spinal cord | Male SD rats | [17] |
| gp120-HNP | / | Heat denaturation of the complex protein structure of gp120 abolished gp120-induced thermal hyperalgesia. And, both thermal hyperalgesia and mechanical allodynia induced by administration of gp120 were attenuated by pretreatment with inhibitors targeting spinal astroglial cells. | / | / | Male SD rats | [18] |
| gp120-HNP | / | The injection of formalin enhances gp120-induced mechanical allodynia, as tested two weeks later. | / | Spinal Cord | Male SD rats | [19] |
| gp120-HNP | TNFα /TNFRI– mtO2–pCREB | HIV gp120 induced overexpression of pC/EBP in the ipsilateral SCDH. It also increased TNFα, TNFRI, mitochondrial superoxide (mtO2), and pCREB. | Neuronal B35 cell line | L4-5 dorsal horn | Male SD rats | [20] |
| gp120-HNP | / | Stimulation with gp120 in acutely isolated lumbar dorsal spinal cords resulted in the release of nitric oxide (NO) along with proinflammatory cytokines. | / | Spinal cord and CSF | Male SD rats | [21] |
| gp120-HNP | / | Intrathecal administration of gp120 upregulates the meningeal gene expression of proinflammatory signals and induces the release of TNF-α, IL-1β, and IL-6 from the meninges in both in vitro and in vivo settings. | Meninges cells | The duramater and the arachnoid layers of the meninges. | Male SD rats | [22] |
| gp120-HNP | Wnt5a-ROR2-MMP2 | Gp120 regulates astrogliosis, which promotes the expression of hyperalgesia and neuropathic pain via IL-1β modulation through a Wnt5a-ROR2-MMP2 axis. | Dorsal horn neurons | L4-L6 lumbar spinal cord | Male C57BL/6 mice | [23] |
| gp120-HNP | Wnt | Wnt3a a β-catenin are up-regulated in the SCDH of HIV-gp120 pain models | / | The L4 and L5 DRG, and lumbar spinal cord | Male C57 BL/6 J mice | [24] |
| gp120-HNP | / | 1-O-acetylgeopyxin A blocks voltage-gated calcium channels and tetrodotoxin-sensitive voltage-gated sodium channels, thereby reversing mechanical allodynia in a model of gp120-induced sensory neuropathy. | DRG sensory neurons | / | Females SD rats | [25] |
| gp120-HNP |  | The modulation of T-type calcium channels by 5bk was further confirmed to reverse mechanical allodynia in rat models of HIV-associated neuropathy. | DRG neurons | / | Male and female SD rats | [26] |
| Perineural gp120+NRTIs- HNP | P2Y12 /MAPK | Exposure of peripheral nerves to HIV gp120+ddC increased mechanical and thermal hyperalgesia through elevated expression of the P2Y12 receptor, heightened levels of proinflammatory cytokines, and enhanced phosphorylation of p38 MAPK in DRG. | DRG satellite glial cells | L4–6 DRGs | Male SD rats | [27] |
| Perineural gp120-HNP | P2Y12 | Downregulation of P2Y12 expression led to reduced levels of GFAP, IL-1β, TNF-α, and p-Akt proteins in the DRG, consequently mitigating mechanical and thermal hyperalgesia in rats treated with gp120. | Astrocyte | DRGs | Male SD rats | [27] |
| Perineural gp120-HNP | P2X3/ERK1/2 | The P2X3 antagonist attenuated hyperalgesia and neuroinflammation by reducing the levels of ERK1/2 phosphorylation in the DRG. | / | L4–6 DRGs | Male SD rats | [28] |
| Perineural gp120-HNP | / | Perineural treatment with HIV-1 gp120 induced persistent mechanical hypersensitivity without altering sensitivity to thermal or cold stimuli. Additionally, it elicited thigmotactic behavior accompanied by decreased intraepidermal nerve fiber density and macrophage infiltration into the peripheral nerve. | Satellite cell and DRG neurons | DRGs, L5 spinal cord, sciatic nerve and hind paw skin | Male Wistar rats | [29] |
| Perineural gp120-HNP | CXCR4/SDF1 | Application of HIV gp120 to the sciatic nerve induced upregulation of TNFa, CXCR4 and SDF1a in both the DRG and the lumbar spinal dorsal horn. | / | L4-5 DRGs or spinal dorsal horn | Male SD rats | [30] |
| Perineural gp120-HNP | GABA/ Wnt5a | The spinal GABAergic tone is reduced in painful HIV pathogenesis, characterized by elevated levels of RO Sand Wnt5a. | / | L4-5 DRG and SDH | Male SD rats | [31] |
| Perineural gp120-HNP | P2X7/ ERK1/2 | The induction of hyperalgesia by gp120 treatment is associated with increased expression of DRG P2X7, IL-1β, and TNF-α receptors, elevated phosphorylation levels of ERK1/2, and reduced expression of IL-10. | DRG neurons | L4–6 DRGs | Male SD rats | [32] |
| Perineural gp120-HNP | P2X7/ERK | Andrographolide can attenuate mechanical and thermal hyperalgesia induced by gp120 by inhibiting the expression of P2X7 receptors in DRGs, reducing the expression of TNFα-R and IL-1β proteins, and inhibiting the activation of the ERK signaling pathway. | / | L4–6 DRGs | Male SD rats | [33] |
| Perineural gp120-HNP | GAD67/GABAergic | Gp120 induced a reduction in GABA immunoreactivity, an increase in signals of mitochondrial superoxide, and upregulated immunoreactivity expression of pCREB and pC/EBPβ in the spinal dorsal horn. | / | L4-5 DRG or spinal dorsal horn | Male SD rats | [34] |
| Perineural gp120-HNP | CXCR4 | IL-10 vectors exhibit anti-nociceptive effects and simultaneously reverse the upregulation of p-p38, TNFα, SDF1α, and CXCR4 induced by gp120 in the spinal dorsal horn and/or DRGs. | / | L4-5 DRG or the spinal cord | Male SD rats | [35] |
| Perineural gp120-HNP | CXCR4 | HSV vectors expressing IL-10 reversed the upregulation of phosphorylated p38MAPK, TNF-α, SCF-1α, and CXCR4 expression. | / | L4-5 DRG and the SDH | Male SD rats | [36] |
| Perineural gp120-HNP | CPEB-mtROS-CBP | Gp120/ddC triggered neuronal CPEB mediated mitochondrial ROS-CBP signal pathway in the SDH. | / | The spinal SDH | Male SD rats | [37] |
| Perineural gp120-HNP | / | TNFα in the spinal cord and the DRG is implicated in peripheral HIV gp120 neuropathic pain. | / | Spinal cord | Male SD rats | [38] |
| Perineural gp120-HNP | / | Mepivacaine consistently alleviated neuropathic pain symptoms induced by gp120, suggesting its potential utility in alleviating clinical HIV neuropathic pain. | / | / | Male SD rats | [39] |
| Perineural gp120-HNP | P2X3 | Nano-curcumin treatment may inhibit P2X3 activation, decrease the sensitizing DRG primary afferents, and alleviate mechanical hyperalgesia and thermal hyperalgesia in gp120-treated rats. | DRG neurons | L4–6 DRGs | Male SD rats | [40] |
| Perineural gp120-HNP | / | Levels of TNFα protein in nerves and the spinal cord remained unchanged in rats displaying allodynia after intraplantar injection of gp120. | / | Sciatic nerves and spinal cord | Female SD rats | [41] |
| Perineural gp120-HNP | / | Allodynia and hyperalgesia were observed following gp120 with notably axonal swelling and increased tumor necrosis factor alpha TNF-α within the sciatic nerve trunk. And intense astrocytic and microglial activation was observed in the spinal cord. | / | Sciatic nerve and spinal cord | Male SD rats | [42] |
| Perineural gp120-HNP | / | Both intrathecal administration of Drp1 antisense ODN and mdivi-1 reversed the upregulation of mitochondrial superoxide in the spinal dorsal horn in the gp120-induced neuropathic pain state. | / | L4-5 DRGs or spinal dorsal horn | Male SD rats | [43] |
| Perineural gp120-HNP | CXCL1 | Schwann cell-derived CXCL1, secreted in response to X4 gp120 exposure, is responsible for macrophage infiltration into peripheral nerves, thereby associated with pain-like behaviors in mice. | Schwann cells; DRG neurons; RAW 264.7 cells | Sciatic nerve and DRG | Wistar/ST rats and male C57BL/6J JmsSlc mice | [44] |
| Perineural gp120-HNP | P2Y12 | uc.48+ siRNA treatment reduced the upregulation of P2Y12 expression and function in DRG neurons, consequently alleviating hyperalgesia in gp120-treated rats. | HEK 293 Cells; DRG neurons | L4–6 DRGs | Male SD rats | [45] |
| Perineural gp120-HNP | CB | Both FAAH inhibitors markedly reduced cold and tactile allodynia with limited anti-hyperalgesic effects. | / | / | Male SD rats | [46] |
| Perineural gp120-NRTIs-HNP | MCP1/CCR2 SDF1/CXCR4 | The functional upregulation of CCR2 and CXCR4 signaling systems following a combination of gp120 and an NRTI is likely to be of central importance in associated HNP. | DRG neurons | Lumbar DRGs | Female SD rats | [47] |
| gp120-NRTIs-HNP | CCL2 | The chemokine CCL2 is significantly expressed in the DRG of rats treated with perineural HIV-gp120 and/or ddC, accompanied by a reduction in intraepidermal nerve fiber density and spinal gliosis. | / | L4-L5 DRGs, sciatic nerve and lumbar spinal cord | Male SD rats | [48] |
| gp120-NRTIs-HNP | Cav2.X | Treatment with gp120+d4T induced upregulation of Cav2.1, Cav2.2, and Cav2.3 in the DRG, leading to nociception induced by Cav2.X agonists. | / | DRGs, and the skin  from the plantar surface of the hind paw | Female and male C57BL/6 mice | [49] |
| gp120-NRTIs-HNP | / | The degree of mechanical hypersensitivity was found to be positively correlated with spinal cord microgliosis in animals treated with gp120 + ddC, but not in those treated with ddC alone. | Microglia | Spinal cord | Male Wistar rats | [50] |
| NRTIs-HNP (Indinavir) |  | Indinavir treatment significantly changed thigmotactic behavior and a significant reduction in hind paw intraepidermal nerve fiber density, and increased expression of phospho-p38 in microglia. | / | L5 spinal cord, L5 DRGs, and glabrous hindpaw skin, | Male Wistar rats | [51] |
| NRTIs-HNP (ddC, d4T) | / | Electrophysiological evidence of significant demyelinating neuropathy was appeared after the start of d4T treatment. Additionally, spontaneous activity in mechanoinsensitive C-nociceptors was observed in both drug-treated groups | / | Sciatic nerve | Male SD rats | [52] |
| NRTIs-HNP (ddC, ddI and d4T) | / | Intradermal or spinal injection of intracellular calcium modulators significantly attenuated and together eliminated ddC and suramin-induced mechanical hypersensitivity. | Saphenous nerve | / | Male SD rats | [53] |
| NRTIs-HNP(d4T) |  | The administration of trkB-Fc chimera or tyrosine kinase inhibitor blocked BDNF-mediated signaling, attenuated the development of mechanical allodynia, and reduced neuronal activity in HNP. | / | Spinal cord | Male C57BL/6J mice | [54] |
| NRTIs-HNP(d4T) |  | After d4T injection, animals exhibited reductions in intraepidermal nerve fiber density and CGRP/IB4 immunoreactivity, along with increases in myelinated and unmyelinated axon diameters, and downregulation of proteins associated with mitochondrial function. | / | Lumbar spinal cord, L5 DRG, sciatic nerve, sural nerve, and glabrous hind paw skin | Male Wistar rats | [55] |
| NRTIs-HNP (ddC) | JNK | JNK3 plays a critical role in regulating ddC neurotoxicity-induced mechanical pain hypersensitivity, while JNK1 is important for activation of c-Jun and GAP-43 as an essential pathway of a regeneration program. | / | L5-L6 DRGs and spinal cord | JNK1-, JNK2-, and JNK3-null mice | [56] |
| NRTIs-HNP (ddC) | CXCR4 | NRTIs produce pain hypersensitivity through the upregulation of CXCR4 signaling in the DRG. | DRG cells | Lumbar DRGs | Female SD rats | [57] |
| NRTIs-HNP (ddC) | PKC-mediated HuD–GAP43 | Systemic administration of ddC increased the expression and phosphorylation of protein kinase C, enzymes highly involved in pain processes, within periaqueductal grey matter. | / | PAG, thalamus and prefrontal cortex | Male CD1 mice | [58] |
| NRTIs-HNP (ddC) | / | DdC systemically to induce mechanical allodynia and induced overexpression of GFAP and TNF-a in the spinal dorsal horn. | HAPI cell | L4/5 DRG | Male SD rats | [59] |
| NRTIs-HNP (ddC) | TNF-α/SDF1/CXCR4 | TNF-α through the SDF1/CXCR4 system is  involved in the NRTIs-related neuropathic pain state and that blocking the signaling of these proinflammatory molecules is able to reduce NRTIs-related neuropathic pain. | / | L4-5 DRG and spinal cord | Male SD rats | [60] |
| NRTIs-HNP (ddC) | Wnt5a | DdC induced notable neuroinflammation in the spinal cord, as evidenced by the upregulation of proinflammatory cytokines TNF-α and IL-1β, along with microglial and astrocytic responses mediated by Wnt5a signaling. | / | Spinal cord | Male C57BL/BL6 mice | [61] |
| NRTIs-HNP (ddC) | 5-HT2A | The 5-HT2A receptor is implicated in both peripheral sensitization of nociceptors and widespread central sensitization of dorsal horn neurons in the HNP model. | DRG cells | Lumbar DRGs | Male Wistar rats; 5-HT2AR-/- mutant mice | [62] |
| NRTIs-HNP (ddC) | / | Gabapentin and morphine, known to reduce mechanical hypersensitivity in these rats, significantly diminish measures of thigmotaxis in the open field. | / | / | Male Wistar rats | [63] |
| NRTIs-HNP (ddC) | HuD–BDNF–NF-H | The presence of a HuD–BDNF–NF-H pathway is activated as a regenerative response to axonal damage induced by ddC treatment, countering the antiretroviral neurotoxicity. | NSC34 cells | Lumbar spinal cord and DRGs | Male CD1 mice | [64] |
| NRTIs-HNP (ddC) | CD11b/p38MAPK | The changes in neuroimmune cells and molecules in the spinal cords during ddC-induced neuroinflammation were sex-dependent, with female mice exhibiting a greater propensity for neuroimmune changes compared to male mice. | / | Spinal cord | Female and male BALB/c mice | [65] |
| NRTIs-HNP (ddC) | GPR55 | DdC treatment resulted in thermal hyperalgesia and increased transcripts of the synthesizing enzyme Plcβ1 and decreased Daglβ in the paw skins, but not Napepld, and Daglα. |  | Brains, spinal cords and paw skins | Female BALB/c mice | [66] |
| NRTIs-HNP (ddC) | CB | BCP prevents NRTI-induced mechanical allodynia, possibly by reducing the inflammatory response, and attenuates mechanical allodynia through CB receptor activation. | / | Half brains and paw skins | Female BALB/c mice | [67] |
| NRTIs-HNP (ddC) | / | DdC caused significant changes in the expression of 135 genes and they are mainly enriched in regulation of transcription, multicellular organism development, and cell differentiation, and the pathway is mainly enriched in the cGMP-PKG signaling pathway and AMPK signaling pathway. | / | Spinal cord | Male C57BL/6J mice | [68] |
| NRTIs-HNP (ddC) | / | Twice-daily intraperitoneal administration of AT2R antagonists yielded significant analgesic effects in NRTIs-HNP. | / | / | Male SD rats | [69] |
| NRTIs-HNP (ddC) | / | DdC neuropathy was mitochondrial-dependent and PKCe-independent, and alcohol-induced painful neuropathy was PKCe-dependent and mitochondrial independent. | / | DRGs | Male SD rats | [70] |
| NRTIs-SIV-HNP | TRPA1/ TRPA1 | SIV-infected and SIVþ/ART animals showed significant increased expression of nociceptive ion channels, TRPV1, and TRPA1 among DRG neurons in SIVþ/ART, and reduced innervation of the nonpeptidergic nociceptors into the dorsal horn. | / | Lumbar spinal cord and DRGs | Indian rhesus macaques | [71] |
| transgenic HNP (Tg) | / | HIV-1Tg rats exhibit similar behavior to those with HNP, specifically, cold sensitivity | / | / | hemizygous HIV-1Tg and non-transgenic Fisher NHsd (F344) rats | [72] |
| transgenic HNP (Tat) | / | Exposure to HIV Tat protein induces hyperalgesia and reduced grooming behavior, which is associated with dynamic changes in oxidative stress, expression of gliotic markers, and integrity of the blood-brain barrier. | / | Whole brain or spinal cord | C57BL/6J mice; iTat bigenic mice; G-tg  transgenic mouse | [73] |
| transgenic HNP (Tat) | / | HIV-1 Tat seems to directly contribute to HIV sensory neuropathy and highlights sex differences in responsiveness to HIV and/or the underlying peripheral neuroinflammatory and nociceptive mechanisms. |  |  | Male and female HIV-Tat transgenic mouse | [74] |
| transgenic HNP (Tat) | / | The Tat protein contributes to the painful sensory neuropathy associated with HIV during the initial stages of pathogenesis. | / | Cervical and lumbar Spinal cord and DRGs, and skin samples | HIV Tat1-86 transgenic mice | [75] |
| transgenic HNP (Tat) | / | There are fundamental sex differences in mechanical and cold hypersensitivity in response to Tat, highlighting the intractable nature of current therapeutics in female mice. | / | Intraepidermal nerve  fibers; lumbar spinal cord and L4-L6 DRGs | Male and female transgenic mice | [76] |
| transgenic HNP (Vpr) | / | Vpr caused DRG neuronal damage, likely through cytosolic calcium activation and cytokine perturbaftion, emphasizing Vpr's role in contributing to HIV-associated peripheral neuropathic pain. | Rat and human DRG | Sural nerves and DRGs | Male SD rats and vpr transgenic mice | [77] |
| transgenic HNP (Vpr) | TrkA | The TrkA receptor agonist indicated that NGF acted through the TrkA receptor to counteract the Vpr-mediated decrease in axon outgrowth in DRG. | Sural nerves; Embryonic human DRGs | L4-L5 DRGs | SD rats; vpr/RAG1-/- mice | [78] |
| Morphine-transgenic (Tg)-ARTs-HNP | / | Opioid- and ART-induced gut microbial dysbiosis, resulting in increased inflammation, may contribute to the exacerbation of the development and persistence of HIV-associated pain in patients. | / | The colon | Male transgenic HIV Tg26 mice of C57BL/6 | [79] |
| Morphine-transgenic HNP(Tat) | / | Tat expression levels can modulate the efficacy of morphine's effects on antinociception and motor coordination/sensorimotor activity. | / | Spinal cord and the striatum | Male DOX-inducible, brain-specific HIV-Tat1-86 transgenic mice; Swiss-Webster mice | [80] |
| Morphine-transgenic HNP(Tat) | / | HIV-1-infected individuals may exhibit heightened analgesic tolerance to similar doses of opiates compared to uninfected individuals, and demonstrate fewer symptoms of physical dependence. | / | / | Doxycycline-inducible, brain-specific HIV-1IIIB Tat1-86 transgenic mice | [81] |
| Morphine-transgenic HNP(Tat) | PPARs | Long-term exposure to Tat decreased specific cytokines and microglial reactivity. However, short-term escalating morphine exposure shifts baseline inflammatory responsiveness. | Microglia | Dorsal striatum and spinal cord | Male Tat1-86 transgenic mice | [82] |
| opioid HNP | Wnt5a | Morphine exacerbates the development of HIV-associated pain, including astroglial activation, pro-inflammatory cytokine expression, and Wnt5a signaling. | Astrocyte | Lumbar spinal dorsal horn | Male C57BL6 mice | [83] |
| opioid HNP | CXCR4 | The analgesic activity of morphine can be reduced by the presence of gp120 and it can be restored by antagonism of CXCR4 receptors | / | Brain periaqueductal grey | Male SD rats | [84] |
| opioid HNP |  | Morphine administration potentiated mitochondrial ROS in the SDH of the HIV pain model. | Astrocytes | Lumbar spinal cord | C57BL6 mice | [85] |
| opioid HNP | Brd4 | The gp120/M induced overexpression of Brd4, and BET inhibitor dose-dependently increased the mechanical threshold in the gp120/M pain state. | / | L4-5 SCDH | Male SD rats | [86] |
| opioid-gp120 HNP | / | Gp120/M increased the expression of spinal TNFRI, mitochondrial superoxide, and cleaved caspase-11. | Neuronal cells | SCDH | Male and female SD rats | [87] |
| opioid-Tat HNP | / | Reduced glial-Beclin1 may provide a layer of protection to neurons when exposed to morphine and Tat | Primary Murine Neuron and Mixed Glia | Frontal cortex | C57BL/6 J and the Becn1 deficient mouse model | [88] |

The table presents potential mechanisms of different HNP models, including information on model types, potential pathways, biofunctions, and other relevant details. “/” means not mentioned in the article. BDNF: Brain-Derived Neurotrophic Factor; ICR mice: Institute of Cancer Research mice; JNK: c-Jun N-terminal Kinase; TNF-a: Tumor Necrosis Factor-alpha; SDH: Succinate Dehydrogenase; P2X7: Purinergic Receptor P2X, Ligand-Gated Ion Channel 7; ERK1/2: Extracellular Signal-Regulated Kinase 1/2; FKN: Fractalkine; CX3R1: CX3C Chemokine Receptor 1; NF-κB: Nuclear Factor Kappa B; CSF: Cerebrospinal Fluid; Ca2+: Calcium ion; AchRs: Acetylcholine Receptors; CREB: cAMP Response Element-Binding Protein; ROR2: Receptor Tyrosine Kinase-Like Orphan Receptor 2; MMP2: Matrix Metalloproteinase-2; IL: Interleukin; GFAP: Glial Fibrillary Acidic Protein; P2Y12: Purinergic Receptor P2Y, G-Protein Coupled 12; Akt: Protein Kinase B; DRG: Dorsal Root Ganglion;P2X3: Purinergic Receptor P2X, Ligand-Gated Ion Channel 3; CXCR4: C-X-C Chemokine Receptor Type 4; SDF1: Stromal Cell-Derived Factor 1; GABA: Gamma-Aminobutyric Acid; CXCL1: C-X-C Motif Chemokine Ligand 1; CCR2: C-C Chemokine Receptor Type 2; CCL2: C-C Motif Chemokine Ligand 2; CGRP: Calcitonin Gene-Related Peptide; IB4: Isolectin B4; NRTI: Nucleoside Reverse Transcriptase Inhibitor; 5-HT2A: 5-Hydroxytryptamine Receptor 2A; MAPK: Mitogen-Activated Protein Kinase; CB: Cannabinoid; GMP: Guanosine Monophosphate; PKG: Protein Kinase G; AMPK: AMP-Activated Protein Kinase; AT2R: Angiotensin II Type 2 Receptor; TRPV1: Transient Receptor Potential Vanilloid 1; TRPA1: Transient Receptor Potential Ankyrin 1; TrkA: Tropomyosin Receptor Kinase A; PPARs: Peroxisome Proliferator-Activated Receptors; Brd4: Bromodomain-Containing Protein 4

**Reference**

1. Zhou X, Tao L, Zhao M, et al. Wnt/β-catenin signaling regulates brain-derived neurotrophic factor release from spinal microglia to mediate HIV1 gp120-induced neuropathic pain. *Molecular Pain*. 2020;16:1-14. doi:10.1177/1744806920922100

2. Yuan SB, Ji G, Li B, Andersson T, Neugebauer V, Tang SJ. A Wnt5a signaling pathway in the pathogenesis of HIV-1 gp120-induced pain. *Pain*. 2015;156(7):1311-1319. doi:10.1097/j.pain.0000000000000177

3. Yuan SB, Shi Y, Chen J, et al. Gp120 in the pathogenesis of human immunodeficiency virus-associated pain. *Annals of Neurology*. 2014;75(6):837-850. doi:10.1002/ana.24139

4. Wu B, Ma Y, Yi Z, et al. Resveratrol-decreased hyperalgesia mediated by the P2X7 receptor in gp120-treated rats. *Molecular Pain*. 2017;13:1-9. doi:10.1177/1744806917707667

5. Guindon J, Blanton H, Brauman S, Donckels K, Narasimhan M, Benamar K. Sex differences in a rodent model of HIV-1-associated neuropathic pain. *International Journal of Molecular Sciences*. 2019;20(5):1-7. doi:10.3390/ijms20051196

6. Shi Y, Shu J, Liang Z, Yuan S, Tang SJ. Oligodendrocytes in HIV-associated pain pathogenesis. *Molecular Pain*. 2016;12:1-7. doi:10.1177/1744806916656845

7. Godai K, Takahashi K, Kashiwagi Y, et al. Ryanodine Receptor to Mitochondrial Reactive Oxygen Species Pathway Plays an Important Role in Chronic Human Immunodeficiency Virus gp120MN-Induced Neuropathic Pain in Rats. *Anesthesia and Analgesia*. 2019;129(1):276-286. doi:10.1213/ANE.0000000000003916

8. Ru W, Liu X, Bae C, et al. Microglia mediate HIV-1 gp120-induced synaptic degeneration in spinal pain neural circuits. *Journal of Neuroscience*. 2019;39(42):8408-8421. doi:10.1523/jneurosci.2851-18.2019

9. Schoeniger-Skinner DK, Ledeboer A, Frank MG, et al. Interleukin-6 mediates low-threshold mechanical allodynia induced by intrathecal HIV-1 envelope glycoprotein gp120. *Brain, Behavior, and Immunity*. 2007;21(5):660-667. doi:10.1016/j.bbi.2006.10.010

10. Palma J, Cowan A, Geller EB, Adler MW, Benamar K. Differential antinociceptive effects of buprenorphine and methadone in the presence of HIV-gp120. *Drug and Alcohol Dependence*. 2011;118(2-3):497-499. doi:10.1016/j.drugalcdep.2011.04.010

11. Ledeboer A, Gamanos M, Lai W, et al. Involvement of spinal cord nuclear factor κB activation in rat models of proinflammatory cytokine-mediated pain facilitation. *European Journal of Neuroscience*. 2005;22(8):1977-1986. doi:10.1111/j.1460-9568.2005.04379.x

12. Huang J, Lin F, Hu Y, Bloe CB, Wang D, Zhang W. From Initiation to Maintenance: HIV-1 Gp120-induced Neuropathic Pain Exhibits Different Molecular Mechanisms in the Mouse Spinal Cord Via Bioinformatics Analysis Based on RNA Sequencing. *Journal of Neuroimmune Pharmacology*. 2022;17(3-4):553-575. doi:10.1007/s11481-021-10044-1

13. Ledeboer A, Sloane EM, Milligan ED, et al. Minocycline attenuates mechanical allodynia and proinflammatory cytokine expression in rat models of pain facilitation. *Pain*. 2005;115(1-2):71-83. doi:10.1016/j.pain.2005.02.009

14. Shanmugam S, Patel D, Guindon J, Reddy PH, Narasimhan M, Benamar K. Gene expression of endocannabinoid system in HIV-1-related neuropathic pain model. *Biochimica et Biophysica Acta - Molecular Basis of Disease*. 2020;1866(10):165891. doi:10.1016/j.bbadis.2020.165891

15. Minami T, Matsumura S, Mabuchi T, et al. Functional evidence for interaction between prostaglandin EP3 and κ-opioid receptor pathways in tactile pain induced by human immunodeficiency virus type-1 (HIV-1) glycoprotein gp120. *Neuropharmacology*. 2003;45(1):96-105. doi:10.1016/S0028-3908(03)00133-3

16. Milligan ED, O’Connor KA, Nguyen KT, et al. Intrathecal HIV-1 envelope glycoprotein gp120 induces enhanced pain states mediated by spinal cord proinflammatory cytokines. *Journal of Neuroscience*. 2001;21(8):2808-2819. doi:10.1523/jneurosci.21-08-02808.2001

17. Loram LC, Harrison JA, Chao L, et al. Intrathecal injection of an alpha seven nicotinic acetylcholine receptor agonist attenuates gp120-induced mechanical allodynia and spinal pro-inflammatory cytokine profiles in rats. *Brain, Behavior, and Immunity*. 2010;24(6):959-967. doi:10.1016/j.bbi.2010.03.008

18. Milligan ED, Mehmert KK, Hinde JL, et al. Thermal hyperalgesia and mechanical allodynia produced by intrathecal administration of the human immunodeficiency virus-1 (HIV-1) envelope glycoprotein, gp120. *Brain Research*. 2000;861(1):105-116. doi:10.1016/S0006-8993(00)02050-3

19. Hains LE, Loram LC, Weiseler JL, et al. Pain intensity and duration can be enhanced by prior challenge: Initial evidence suggestive of a role of microglial priming. *Journal of Pain*. 2010;11(10):1004-1014. doi:10.1016/j.jpain.2010.01.271

20. Yi H, Liu S, Kashiwagi Y, et al. Phosphorylated CCAAT/enhancer binding protein β Contributes to rat HIV-related neuropathic pain: In vitro and in vivo studies. *Journal of Neuroscience*. 2018;38(3):555-574. doi:10.1523/JNEUROSCI.3647-16.2017

21. Holguin A, O’Connor KA, Biedenkapp J, et al. HIV-1 gp120 stimulates proinflammatory cytokine-mediated pain facilitation via activation of nitric oxide synthase-I (nNOS). *Pain*. 2004;110(3):517-530. doi:10.1016/j.pain.2004.02.018

22. Wieseler-Frank J, Jekich BM, Mahoney JH, Bland ST, Maier SF, Watkins LR. A novel immune-to-CNS communication pathway: Cells of the meninges surrounding the spinal cord CSF space produce proinflammatory cytokines in response to an inflammatory stimulus. *Brain, Behavior, and Immunity*. 2007;21(5):711-718. doi:10.1016/j.bbi.2006.07.004

23. Liu X, Bae C, Gelman BB, Chung JM, Tang SJ. A neuron-to-astrocyte Wnt5a signal governs astrogliosis during HIV-associated pain pathogenesis. *Brain*. 2022;145(11):4108-4123. doi:10.1093/brain/awac015

24. Shi Y, Yuan S, Li B, et al. Regulation of Wnt signaling by nociceptive input in animal models. *Molecular Pain*. 2012;8(1):1. doi:10.1186/1744-8069-8-47

25. Zhou Y, Cai S, Gomez K, et al. 1-O-Acetylgeopyxin A, a derivative of a fungal metabolite, blocks tetrodotoxin-sensitive voltage-gated sodium, calcium channels and neuronal excitability which correlates with inhibition of neuropathic pain. *Molecular Brain*. 2020;13(1):1-12. doi:10.1186/s13041-020-00616-2

26. Cai S, Tuohy P, Ma C, et al. A modulator of the low-voltage-activated T-type calcium channel that reverses HIV glycoprotein 120-, paclitaxel-, and spinal nerve ligation-induced peripheral neuropathies. *Pain*. 2020;161(11):2551-2570. doi:10.1097/j.pain.0000000000001955

27. Yi Z, Xie L, Zhou C, et al. P2Y12 receptor upregulation in satellite glial cells is involved in neuropathic pain induced by HIV glycoprotein 120 and 2′,3′-dideoxycytidine. *Purinergic Signalling*. 2018;14(1):47-58. doi:10.1007/s11302-017-9594-z

28. Yi Z, Rao S, Ouyang S, et al. A317491 relieved HIV gp120-associated neuropathic pain involved in P2X3 receptor in dorsal root ganglia. *Brain Research Bulletin*. 2017;130:81-89. doi:10.1016/j.brainresbull.2017.01.002

29. Wallace VCJ, Blackbeard J, Pheby T, et al. Pharmacological, behavioural and mechanistic analysis of HIV-1 gp120 induced painful neuropathy. *Pain*. 2007;133(1-3):47-63. doi:10.1016/j.pain.2007.02.015

30. Huang W, Zheng W, Liu S, et al. HSV-mediated p55TNFSR reduces neuropathic pain induced by HIV gp120 in rats through CXCR4 activity. *Gene Therapy*. 2014;21(3):328-336. doi:10.1038/gt.2013.90

31. Kanda H, Kanao M, Liu S, et al. HSV vector-mediated GAD67 suppresses neuropathic pain induced by perineural HIV gp120 in rats through inhibition of ROS and Wnt5a. *Gene Therapy*. 2016;23(4):340-348. doi:10.1038/gt.2016.3

32. Wu B, Peng L, Xie J, et al. The P2X7 receptor in dorsal root ganglia is involved in HIV gp120-associated neuropathic pain. *Brain Research Bulletin*. 2017;135(July 2016):25-32. doi:10.1016/j.brainresbull.2017.09.006

33. Yi Z, Ouyang S, Zhou C, et al. Andrographolide inhibits mechanical and thermal hyperalgesia in a rat model of HIV-induced neuropathic pain. *Frontiers in Pharmacology*. 2018;9(JUN):1-12. doi:10.3389/fphar.2018.00593

34. Kanao M, Kanda H, Huang W, et al. Gene transfer of glutamic acid decarboxylase 67 by herpes simplex virus vectors suppresses neuropathic pain induced by human immunodeficiency virus gp120 combined with ddC in rats. *Anesthesia and Analgesia*. 2015;120(6):1394-1404. doi:10.1213/ANE.0000000000000729

35. Zheng W, Huang W, Liu S, et al. IL-10 mediated by herpes simplex virus vector reduces neuropathic pain induced by HIV gp120 combined with ddC in rats. *Molecular Pain*. 2014;10(1):1-12. doi:10.1186/1744-8069-10-49

36. Zheng W, Huang W, Liu S, et al. Interleukin 10 mediated by herpes simplex virus vectors suppresses neuropathic pain induced by human immunodeficiency virus gp120 in rats. *Anesthesia and Analgesia*. 2014;119(3):693-701. doi:10.1213/ANE.0000000000000311

37. Iida T, Yi H, Liu S, et al. Spinal CPEB-mtROS-CBP signaling pathway contributes to perineural HIV gp120 with ddC-related neuropathic pain in rats. *Experimental Neurology*. 2016;281:17-27. doi:10.1016/j.expneurol.2016.04.012

38. Zheng W, Ouyang H, Zheng X, et al. Glial TNFα in the spinal cord regulates neuropathic pain induced by HIV gp120 application in rats. *Molecular Pain*. 2011;7(1):40. doi:10.1186/1744-8069-7-40

39. Sagen J, Castellanos DA, Hama AT. Antinociceptive effects of topical mepivacaine in a rat model of HIV-associated peripheral neuropathic pain. *Journal of Pain Research*. 2016;9:361-371. doi:10.2147/JPR.S104397

40. Zhao S, Yang J, Han X, et al. Effects of nanoparticle-encapsulated curcumin on HIV-gp120-associated neuropathic pain induced by the P2X3 receptor in dorsal root ganglia. *Brain Research Bulletin*. 2017;135(May):53-61. doi:10.1016/j.brainresbull.2017.09.011

41. Jolivalt CG, daCunha JM, Esch FS, Calcutt NA. Central action of prosaptide TX14(A) against gp120-induced allodynia in rats. *European Journal of Pain*. 2008;12(1):76-81. doi:10.1016/j.ejpain.2007.03.008

42. Herzberg U, Sagen J. Peripheral nerve exposure to HIV viral envelope protein gp120 induces neuropathic pain and spinal gliosis. *Journal of Neuroimmunology*. 2001;116(1):29-39. doi:10.1016/S0165-5728(01)00288-0

43. Kanda H, Liu S, Iida T, et al. Inhibition of Mitochondrial Fission Protein Reduced Mechanical Allodynia and Suppressed Spinal Mitochondrial Superoxide Induced by Perineural Human Immunodeficiency Virus gp120 in Rats. *Anesthesia and Analgesia*. 2016;122(1):264-272. doi:10.1213/ANE.0000000000000962

44. Ntogwa M, Imai S, Hiraiwa R, et al. Schwann cell-derived CXCL1 contributes to human immunodeficiency virus type 1 gp120-induced neuropathic pain by modulating macrophage infiltration in mice. *Brain, Behavior, and Immunity*. 2020;88:325-339. doi:10.1016/j.bbi.2020.03.027

45. Peng L, Wu B, Shi L, et al. Long Non-coding RNA Uc.48+ Small Interfering RNA Alleviates Neuroinflammatory Hyperalgesia in Gp120-Treated Rats via the P2Y12 Receptor. *Frontiers in Neuroscience*. 2021;15(July):1-10. doi:10.3389/fnins.2021.663962

46. Nasirinezhad F, Jergova S, Pearson JP, Sagen J. Attenuation of persistent pain-related behavior by fatty acid amide hydrolase (FAAH) inhibitors in a rat model of HIV sensory neuropathy. *Neuropharmacology*. 2015;95:100-109. doi:10.1016/j.neuropharm.2014.11.024

47. Bhangoo SK, Ripsch MS, Buchanan DJ, Miller RJ, White FA. Increased chemokine signaling in a model of HIV1-associated peripheral neuropathy. *Molecular Pain*. 2009;5:1-18. doi:10.1186/1744-8069-5-48

48. Wallace VCJ, Blackbeard J, Segerdahl AR, et al. Characterization of rodent models of HIV-gp120 and anti-retroviral- associated neuropathic pain. *Brain*. 2007;130(10):2688-2702. doi:10.1093/brain/awm195

49. Lückemeyer DD, Prudente AS, de Amorim Ferreira M, et al. Critical Pronociceptive Role of Family 2 Voltage-Gated Calcium Channels in a Novel Mouse Model of HIV-Associated Sensory Neuropathy. *Molecular Neurobiology*. 2023;60(5):2954-2968. doi:10.1007/s12035-023-03244-8

50. Blackbeard J, Wallace VCJ, O’Dea KP, et al. The correlation between pain-related behaviour and spinal microgliosis in four distinct models of peripheral neuropathy. *European Journal of Pain (United Kingdom)*. 2012;16(10):1357-1367. doi:10.1002/j.1532-2149.2012.00140.x

51. Huang W, Calvo M, Pheby T, Bennett DLH, Rice ASC. *A Rodent Model of HIV Protease Inhibitor Indinavir Induced Peripheral Neuropathy*. Vol 158.; 2017. doi:10.1097/j.pain.0000000000000727

52. Garcia-perez E, Sumalla M, Serra J. Behavioral and electrophysiological abnormalities in two rat models of antiretroviral. 2015;156(9).

53. Joseph EK, Chen X, Khasar SG, Levine JD. Novel mechanism of enhanced nociception in a model of AIDS therapy-induced painful peripheral neuropathy in the rat. *Pain*. 2004;107(1-2):147-158. doi:10.1016/j.pain.2003.10.010

54. Renn CL, Leitch CC, Lessans S, et al. Brain-Derived Neurotrophic Factor Modulates Antiretroviral-Induced Mechanical Allodynia in the Mouse. 2011;1565:1551-1565. doi:10.1002/jnr.22685

55. Huang W, Calvo M, Karu K, et al. A clinically relevant rodent model of the HIV antiretroviral drug stavudine induced painful peripheral neuropathy. *Pain*. 2013;154(4):560-575. doi:10.1016/j.pain.2012.12.023

56. Sanna MD, Manassero G, Vercelli A, Herdegen T, Galeotti N. The isoform-specific functions of the c-Jun N-terminal kinase (JNK) in a mouse model of antiretroviral-induced painful peripheral neuropathy. *European Journal of Pharmacology*. 2020;880:173161. doi:10.1016/j.ejphar.2020.173161

57. Bhangoo SK, Ren D, Miller RJ, et al. CXCR4 chemokine receptor signaling mediates pain hypersensitivity in association with antiretroviral toxic neuropathy. *Brain, Behavior, and Immunity*. 2007;21(5):581-591. doi:10.1016/j.bbi.2006.12.003

58. Sanna MD, Quattrone A, Ghelardini C, Galeotti N. PKC-mediated HuD-GAP43 pathway activation in a mouse model of antiretroviral painful neuropathy. *Pharmacological Research*. 2014;81:44-53. doi:10.1016/j.phrs.2014.02.004

59. Zheng X, Ouyang H, Liu S, Mata M, Fink DJ, Hao S. TNFα is involved in neuropathic pain induced by nucleoside reverse transcriptase inhibitor in rats. *Brain, Behavior, and Immunity*. 2011;25(8):1668-1676. doi:10.1016/j.bbi.2011.06.010

60. Huang W, Zheng W, Ouyang H, et al. Mechanical allodynia induced by nucleoside reverse transcriptase inhibitor is suppressed by p55TNFSR mediated by herpes simplex virus vector through the SDF1 alpha/CXCR4 system in rats. *Anesthesia and Analgesia*. 2014;118(3):671-680. doi:10.1213/ANE.0000000000000079

61. Yuan S, Shi Y, Guo K, Tang SJ. Nucleoside Reverse Transcriptase Inhibitors (NRTIs) Induce Pathological Pain through Wnt5a-Mediated Neuroinflammation in Aging Mice. *Journal of Neuroimmune Pharmacology*. 2018;13(2):230-236. doi:10.1007/s11481-018-9777-6

62. Van Steenwinckel J, Brisorgueil MJ, Fischer J, et al. Role of spinal serotonin 5-HT2A receptor in 2′,3′-dideoxycytidine-induced neuropathic pain in the rat and the mouse. *Pain*. 2008;137(1):66-80. doi:10.1016/j.pain.2007.08.014

63. Wallace VCJ, Segerdahl AR, Blackbeard J, Pheby T, Rice ASC. Neuroscience Letters Anxiety-like behaviour is attenuated by gabapentin , morphine and diazepam in a rodent model of HIV anti-retroviral-associated neuropathic pain. 2008;448:153-156. doi:10.1016/j.neulet.2008.10.005

64. Sanna MD, Peroni D, Mello T, Ghelardini C, Quattrone A, Galeotti N. Increase of neurofilament-H protein in sensory neurons in antiretroviral neuropathy : Evidence for a neuroprotective response mediated by the RNA-binding protein HuD. *Pharmacological Research*. 2016;111:23-33. doi:10.1016/j.phrs.2016.05.026

65. Al-HadlaQ MW, Masocha W. Sex Differences in the Expression of Neuroimmune Molecules in the Spinal Cord of a Mouse Model of Antiretroviral-Induced Neuropathic Pain. *Biomedicines*. 2023;11(3). doi:10.3390/biomedicines11030875

66. Munawar N, Oriowo MA, Masocha W. Antihyperalgesic activities of endocannabinoids in a mouse model of antiretroviral-Induced neuropathic pain. *Frontiers in Pharmacology*. 2017;8(MAR):1-11. doi:10.3389/fphar.2017.00136

67. Esraa A, Maitham K, Willias M. β-Caryophyllene, a CB2-Receptor-Selective Phytocannabinoid, Suppresses Mechanical Allodynia in a Mouse Model of Antiretroviral-Induced Neuropathic Pain. *Molecules*. 2020;25(106):1-20.

68. Wu S, Yang S, Bloe CB, Zhuang R, Huang J, Zhang W. Identification of Key Genes and Pathways in Mouse Spinal Cord Involved in ddC-Induced Neuropathic Pain by Transcriptome Sequencing. *Journal of Molecular Neuroscience*. 2021;71(3):651-661. doi:10.1007/s12031-020-01686-6

69. Smith MT, Lau T, Wallace VCJ, Wyse BD, Rice ASC. Analgesic efficacy of small-molecule angiotensin II type 2 receptor antagonists in a rat model of antiretroviral toxic polyneuropathy. *Behavioural Pharmacology*. 2014;25(2):137-146. doi:10.1097/FBP.0000000000000025

70. Ferrari LF, Levine JD. Alcohol consumption enhances antiretroviral painful peripheral neuropathy by mitochondrial mechanisms. *European Journal of Neuroscience*. 2010;32(5):811-818. doi:10.1111/j.1460-9568.2010.07355.x

71. Bravo-Caparrós I, Ruiz-Cantero MC, Perazzoli G, et al. Sigma-1 receptors control neuropathic pain and macrophage infiltration into the dorsal root ganglion after peripheral nerve injury. *FASEB Journal*. 2020;34(4):5951-5966. doi:10.1096/fj.201901921R

72. War R, Robinson JA, Podgorski RM, Miller AD, Burdo TH. Neuroin fl ammation in the Dorsal Root Ganglia and Dorsal Horn Contributes to Persistence of Nociceptor Sensitization in SIV-Infected Antiretroviral Therapy e Treated Macaques. 2023;(September):1-14. doi:10.1016/j.ajpath.2023.08.014

73. E Gryshyna A, Chatterjee T, J DeBerry J, Aggarwal S. Assessment of pain-related behaviors in HIV-1 transgenic rats as a model of HIV-associated chronic pain. *Molecular Pain*. 2023;19:1-10. doi:10.1177/17448069231213554

74. Bagdas D, Paris JJ, Carper M, et al. Conditional expression of HIV-1 tat in the mouse alters the onset and progression of tonic, inflammatory and neuropathic hypersensitivity in a sex-dependent manner. *European Journal of Pain (United Kingdom)*. 2020;24(8):1609-1623. doi:10.1002/ejp.1618

75. Wodarski R, Bagdas D, Paris JJ, et al. Reduced intraepidermal nerve fibre density, glial activation, and sensory changes in HIV type-1 Tat-expressing female mice: Involvement of Tat during early stages of HIV-associated painful sensory neuropathy. *Pain Reports*. 2018;3(3):1-12. doi:10.1097/PR9.0000000000000654

76. Toma W, Paris JJ, Warncke UO, et al. Persistent sensory changes and sex differences in transgenic mice conditionally expressing HIV-1 Tat regulatory protein. *Experimental Neurology*. 2022;358(August):114226. doi:10.1016/j.expneurol.2022.114226

77. Acharjee S, Noorbakhsh F, Stemkowski PL, et al. HIV‐1 viral protein R causes peripheral nervous system injury associated with in vivo neuropathic pain . *The FASEB Journal*. 2010;24(11):4343-4353. doi:10.1096/fj.10-162313

78. Webber CA, Salame J, Luu GLS, et al. Nerve growth factor acts through the TrkA receptor to protect sensory neurons from the damaging effects of the HIV-1 viral protein, Vpr. *Neuroscience*. 2013;252:512-525. doi:10.1016/j.neuroscience.2013.07.046

79. Antoine D, Chupikova I, Jalodia R, Singh PK, Roy S. Chronic Morphine Treatment and Antiretroviral Therapy Exacerbate HIV-Distal Sensory Peripheral Neuropathy and Induce Distinct Microbial Alterations in the HIV Tg26 Mouse Model. *International Journal of Molecular Sciences*. 2024;25(3). doi:10.3390/ijms25031569

80. Fitting S, Scoggins KL, Xu R, et al. Morphine efficacy is altered in conditional HIV-1 Tat transgenic mice. *European Journal of Pharmacology*. 2012;689(1-3):96-103. doi:10.1016/j.ejphar.2012.05.029

81. Fitting S, Stevens DL, Khan FA, et al. Morphine Tolerance and Physical Dependence Are Altered in Conditional HIV-1 Tat Transgenic Mice. *The Journal of pharmacology and experimental therapeutics*. 2016;356(1):96-105. doi:10.1124/jpet.115.226407

82. Hermes DJ, Jacobs IR, Key MC, et al. Escalating morphine dosing in HIV-1 Tat transgenic mice with sustained Tat exposure reveals an allostatic shift in neuroinflammatory regulation accompanied by increased neuroprotective non-endocannabinoid lipid signaling molecules and amino acids. *Journal of Neuroinflammation*. 2020;17(1):1-26. doi:10.1186/s12974-020-01971-6

83. Shi Y, Yuan S, Tang SJ. Morphine and HIV-1 gp120 cooperatively promote pathogenesis in the spinal pain neural circuit. *Molecular Pain*. 2019;15:1-11. doi:10.1177/1744806919868380

84. Chen X, Kirby LG, Palma J, et al. The effect of gp120 on morphine’s antinociceptive and neurophysiological actions. *Brain, Behavior, and Immunity*. 2011;25(7):1434-1443. doi:10.1016/j.bbi.2011.04.014

85. Shi Y, Yuan S, Tang SJ. Reactive Oxygen Species (ROS) are Critical for Morphine Exacerbation of HIV-1 gp120-Induced Pain. *Journal of Neuroimmune Pharmacology*. 2021;16(3):581-591. doi:10.1007/s11481-020-09951-6

86. Takahashi K, Yi H, Liu CH, et al. Spinal bromodomain-containing protein 4 contributes to neuropathic pain induced by HIV glycoprotein 120 with morphine in rats. *NeuroReport*. 2018;29(6):441-446. doi:10.1097/WNR.0000000000000992

87. Hayashi K, Yi H, Zhu X, et al. Role of Tumor Necrosis Factor Receptor 1 - Reactive Oxygen Species - Caspase 11 Pathway in Neuropathic Pain Mediated by HIV gp120 with Morphine in Rats. *Anesthesia and Analgesia*. 2023;136(4):789-801. doi:10.1213/ANE.0000000000006335

88. Lapierre J, Karuppan MKM, Perry M, Rodriguez M, El-Hage N. Different Roles of Beclin1 in the Interaction Between Glia and Neurons after Exposure to Morphine and the HIV- Trans-Activator of Transcription (Tat) Protein. *Journal of Neuroimmune Pharmacology*. 2022;17(3-4):470-486. doi:10.1007/s11481-021-10017-4
