# Supplementary material for: Exploring viral neuropathic pain: Molecular mechanisms and therapeutic implications
Source: PLoS Pathog. 2024 Aug 8;20(8):e1012397. doi: 10.1371/journal.ppat.1012397 (PMC11309435; doi:10.1371/journal.ppat.1012397)
Supplement: S3 Table — The table presents potential mechanisms of PHN models, including information on model types, potential pathways, biofunctions, and other relevant details. “/” means not mentioned in the article. CCL5, C-C motif chemokine ligand 5; CCR5, C-C chemokine receptor type 5; HSV-1, herpes simplex virus type 1; DRG, dorsal root ganglion; TLR4, toll-like receptor 4; TNF, tumor necrosis factor; PHP, pseudohypoparathyroidism; K+, potassium ion; COX, cyclooxygenase; PGE2, prostaglandin E2; EP3, prostaglandin E2 receptor EP3; NMDA, N-Methyl-D-Aspartate; NR2Bs, N-Methyl-D-Aspartate receptor subunit 2B; NOS, nitric oxide synthase; Prmt6, protein arginine methyltransferase 6; cGAS, cyclic GMP-AMP synthase; STING, stimulator of interferon genes; VPM, ventral posterior medial nucleus; VPL, ventral posterior lateral nucleus; P2X7, purinergic receptor P2X, ligand-gated ion channel 7; BBG, brilliant blue G; ER stress, endoplasmic reticulum stress; KCNA2, potassium voltage-gated channel subfamily A member 2; STAT3, signal transducer and activator of transcription 3; pSTAT3, phosphorylated signal transducer and activator of transcription 3; MRC-5, medical research council cell strain 5; TRPV1, transient receptor potential vanilloid 1; NO, nitric oxide; vHPPE, ventriculoperitoneal hydrocephalus; VZV, varicella zoster virus; SNT, syntrophin; H3, histone H3; PWL, paw withdrawal latency; MWT, mechanical withdrawal threshold; TRX, thioredoxin; NP, nucleoside phosphorylase; DNF, brain-derived neurotrophic factor; ASIC3, acid-sensing ion channel 3; PAQR, progestin and AdipoQ receptor; VGAT, vesicular GABA transporter; ERK, extracellular signal-regulated kinase; IE4, immediate early protein 4. (DOCX) [file ppat.1012397.s003.docx]

S3 Table. Potential Molecular Mechanisms in different PHN models

| Model | Receptor/pathway | Biofunctions | Cells | Tissue | Species | Ref. |
| --- | --- | --- | --- | --- | --- | --- |
| HSV‐1 | CCL5/CCR5 | The inhibition of CCR5 demonstrated a significant analgesic effect and effectively alleviated the increase of inflammatory cytokines in both the dorsal root ganglia and spinal cord induced by HSV-1 infection in mice. | / | DRG and spinal cord | Male C57BL/6J mice | [1] |
| HSV‐1 | TLR4/TNF | The pronociceptive function of S100A9 relies on the TLR4/TNF pathway in herpetic neuralgia. | / | L3-L5 DRGs and spinal cord | Male C57BL/6; S100a9 and Tlr4 deficient (−/−) mice | [2] |
| HSV‐1 | / | Amenamevir effectively suppresses the development of AHP and the transition to PHP by reducing viral load in the DRG and decreasing the expression of pain-related genes in the spinal cord, respectively. | / | L4–L6 spinal cord and DRGs | Female C57BL/6 j mice | [3] |
| HSV‐1 | / | Fosphenytoin suppressed HSV-1-induced spontaneous pain-like behaviors. | / | / | Female C57BL/6J mice | [4] |
| HSV‐1 | TNFR1 | Leukocytes infiltrate infected sensory ganglia and play a crucial role in driving the production of TNF, which facilitates the development of herpetic neuralgia by downregulating the inwardly rectifying K channel Kir4.1 in satellite glial cells. | / | L3-L6 DRGs | Male C57BL/6 or BALB/C WT mice | [5] |
| HSV‐1 | PGE2/eEP3 | COX-2 induction and the PGE2-EP3 receptor system play an essential role in the dorsal root ganglia specifically in the development, but not maintenance, of acute herpetic pain. | / | L4- L5 DRGs | Female BALB/c mice; male C57BL/6J mice; prostanoid receptor gene_x005f deficient mice | [6] |
| HSV‐1 | Galectin-3 | Galectin-3 expressed in infiltrating macrophages and/or resident microglia in the spinal dorsal horn contributes to the development of herpetic allodynia. | / | Lumbar spinal dorsal horn | Female C57BL/6J mice; Female galectin-3 gene-deficient mice | [7] |
| HSV‐1 | / | Gabapentin demonstrates efficacy in treating acute herpetic pain with minimal observable adverse effects. Its analgesic effects primarily occur through actions on the spinal cord. | / | / | Female BALB/c mice | [8] |
| HSV‐1 | / | Gabapentin demonstrated a dose-dependent inhibition of acute herpetic pain-related responses. This medication notably reduced the incidence of delayed postherpetic pain. | / | / | Female BALB/c mice | [9] |
| HSV‐1 | / | The non-narcotic µ-opioid receptor agonist loperamide may alleviate acute herpetic pain in patients suffering from herpes zoster. | / | / | Female C57BL/6j mice | [10] |
| HSV‐1 | NMDA/NR2Bs | Phosphorylation of the NR2B subunit at Tyr1472 is implicated in the development of postherpetic allodynia resulting from nerve damage. Furthermore, nerve damage during the acute herpetic phase is associated with the occurrence of postherpetic pain. | DRG neuron | L4-L5 spinal cord and DRGs | C57BL/6 mice; Y1472F-KI mice | [11] |
| HSV‐1 | / | Static allodynia was not prominently observed during the stage of herpetic pain and instead manifested gradually after the healing of the lesion. | / | L5 DRG | Female C57BL/6J mice | [12] |
| HSV‐1 | / | In herpetic mice, the excitatory response of wide-dynamic range neurons to brush stimulation of the zosteriform dermatome increased, while responses to punctum and pinch stimuli remained unchanged. | / | Tibial nerve; spinal cord | Female C57BL/6j mice | [13] |
| HSV‐1 | / | Artemin exerts antinociceptive effects on herpes simplex virus (HSV)-related pain through alterations in dynorphin levels within the central nervous system of HSV-inoculated mice. | / | Mid-brain and hypothalamus; spinal cord | Recombinant mouse AR; BALB/c female mice, | [14] |
| HSV‐1 | / | Sympathetic nerves and adrenoceptors do not participate in the pain-related responses elicited by herpetic infection, | / | / | Female BALB/c mice | [15] |
| HSV‐1 | / | The MHC haplotype (H-2b) is implicated in the occurrence of postherpetic pain, with CD3-positive T cells potentially contributing to its pathogenesis. | / | DRG | Female BALB/c and C57BL/6 mice | [16] |
| HSV‐1 | / | Mexiletine exerts antinociceptive effects on herpes-related pain through the enhancement of β-endorphin levels in the central nervous system in HSV-inoculated mice. | / | Mid brain and hypothalamus | BALB/c female mice | [17] |
| HSV‐1 | NOS1 / NOS2 | Herpetic and postherpetic allodynia are mediated by nitric oxide in the dorsal horn, with NOS2 and NOS1 being responsible for herpetic and postherpetic allodynia. | / | Lumbar dorsal horn | Female C57BL/6j mice | [18] |
| HSV‐1 | / | Viral inoculation induced cutaneous lesions and pain-related responses starting on day 5 post-inoculation. | / | / | Female BALB/c mice | [19] |
| HSV-1 | / | The propagation of HSV-1 in the dorsal root ganglia produces allodynia and hyperalgesia as a result of functional abnormality of the sensory neurons in mice. | / | L4-L5 DRGs | Female BALB/c mice | [20] |
| HSV-1 | Prmt6 | HSV-1 escapes antiviral innate immunity and results in PHN by upregulating Prmt6 expression and inhibiting cGAS-STING pathway | Primary microglia and BV-2 cell culture | Spinal dorsal  horn | Male C57BL/6J mice | [21] |
| left trigeminal ganglion and whisker pad VZV injection model | / | The pain associated with VZV infection doesn't solely stem from mechanisms localized to peripheral nerve terminals and it can be regulated by excitatory neurons within the VPM/VPL. | / | Trigeminal ganglion | Male SD rats | [22] |
| Plantar injection model | P2X7 | The P2X7 receptor antagonist BBG mitigates PHN by triggering ER stress activation and diminishing pyroptosis. | / | L4-L6 spinal cord and DRGs | Wistar rats | [23] |
| Plantar VZV injection model | KCNA2/STAT3 | The alleviation of PHN through down-regulation of KCNA2-AS is partially attributed to the reduction in the cytoplasm-to-nucleus translocation of pSTAT3, subsequently leading to the inhibition of spinal astrocyte activation. | astrocytes | Spinal cord | Female SD rats | [24] |
| Plantar VZV injection model | NMDA | VZV infection induced an increased behavioral reflex responsiveness to both noxious thermal and mechanical stimuli ipsilateral to injection by spinal NMDA receptors, accompanied by upregulation of sodium-calcium channels and ATF-3 expression. | MRC-5 human diploid fibroblast cells | L4-L6 DRGs | Male Wistar rats | [25] |
| Plantar VZV injection model | / | Systemically administered L-29 reduced mechanical hypersensitivity in a model of VZV-associated pain. | / | / | Male Wistar rats | [26] |
| Plantar VZV injection model | TRPV1 | HSV-2-secreted glycoprotein G alters thermal pain sensitivity via modulating TRPV1 receptor. | DRG neurons | Hind paw skin | Male CD-1 mice | [27] |
| Plantar VZV injection model | NMDAR | In the VZV model, NO triggers spinal astrocytic activation. Activated astrocytes then upregulate IL-1βexpression, leading to NMDAR phosphorylation in spinal dorsal horn neurons, intensifying pain transmission. | / | L5 spinal cord | Male Wistar rats | [28] |
| Plantar VZV injection model | / | Subsequent footpad administration of vHPPE attenuated VZV-induced pain behaviors in a dose-dependent manner over prolonged durations, while prophylactic vector administration effectively prevented the development of VZV-induced pain. | / | L4-6 DRGs | Male SD rats | [29] |
| Plantar VZV injection model | / | Infection with all viral strains was linked to dose-dependent mechanical hypersensitivity, accompanied by anxiety-like behavior. | / | L4-L5 DRGs | Male Wistar rats | [30] |
| Plantar VZV injection model | / | Inoculation of rats with VZV results in a single round of incomplete infection, which is adequate to induce pain behaviors, involving infection and induced changes in neuronal populations. | DRG neurons etc. | Footpad skin; DRG | Rats | [31] |
| Plantar VZV injection model | / | spinal microgliosis, was evident in the SNT and to a lesser extent in the HIV neuropathy models but not the VZV model. | / | L5 spinal cord | Male Wistar rats | [32] |
| Plantar VZV injection model | H3 | Chronic oral administration of selective H3 antagonists has been demonstrated to effectively reverse neuropathic hypersensitivity in VZV pain models. | / | Spinal dorsal horn | Male Random-hooded rats | [33] |
| Plantar VZV injection model | estrogen receptor | Estradiol enhances the activity of inhibitory neurons in the thalamus to alleviate herpes zoster pain. | / | Thalamic tissue | Male SD rats | [34] |
| TRX-induced PHN | MECP2 | miR-199-3p improved PWL and MWT, while concurrently modulating the inflammatory response by targeting MECP2, thus regulating TRX-induced pain. | 293T cells | Serum | Male C57BL/6 J mice | [35] |
| TRX-induced PHN | / | Oral TTX pellets were notably effective in preventing RTX-induced mechanical and thermal allodynia, showing comparable efficacy to pregabalin. | / | Plasma | SD rats | [36] |
| TRX-induced PHN | ASIC3 | Inhibiting BDNF/TrkB.T1 reduces inflammation, decreases neuronal hyperexcitability, and improves mechanical allodynia by regulating the ASIC3 signaling pathway in DRGs. | PC-12 cells | L4–L6 DRG | Male SD rats | [37] |
| whisker pad VZV injection model | / | Aversive behavior remained significantly elevated for up to 7 weeks in rats injected with VZV into the whisker pad, exhibiting a sex difference. | MeWo cells | Whisker pad tissue；trigeminal ganglia | Male and female SD or Long Evans rats | [38] |
| whisker pad VZV injection | PAQR8/PAQR9 | The expression of PAQR8 and PAQR9 is modulated by VZV injection, and these alterations are influenced by age. | / | Ventral tegmental tissue | Long Evans male rats | [39] |
| whisker pad VZV injection | VGAT/ERK | Aromatase-derived estradiol interacts with ER to enhance VGAT expression and neuronal inhibition in the thalamus, thereby alleviating VZV-induced pain. | / | Thalamic tissue | Male SD rats | [40] |
| whisker pad VZV injection | IE4/IE63 | It suggests a mechanism for pain induction involving the early expression of IE4 or IE63 proteins in abortively infected neurons after herpes zoster, potentially leading to aberrant host pain signaling and the development of PHN. | / | L4-L6 DRG | Male SD rats | [41] |
| whisker pad VZV injection | / | Estradiol mitigates herpes zoster pain by enhancing the activity of inhibitory neurons within the reticular thalamus, subsequently inhibiting excitatory activity in the ventral posteromedial nucleus, leading to a decrease in orofacial pain. | / | Reticular thalamic nucleus | Transgenic male and female Long Evans rats | [42] |
| whisker pad VZV injection | / | The lateral thalamus controls nociception in the orofacial region, and GABA in this area appears to reduce the response to VZV-induced nociception possibly by gating facial pain input. | / | Lateral thalamic region | Male SD rats | [43] |
| Paraspinal subcutaneous VZV injection | / | Following the establishment of chronic varicella zoster virus infection in rats, behavioral allodynia and hyperalgesia were observed in the injected animals for up to 33 days post infection. | / | Lumbar DRGs | Male Wistar rats | [44] |
| Paraspinal subcutaneous VZV injection | / | The presence of viral material in dissociated and cultured DRG from inoculated animals was investigated using immune-peroxidase and in situ hybridization techniques. | DRG neurons | / | Rats | [45] |

The table presents potential mechanisms of PHN models, including information on model types, potential pathways, biofunctions, and other relevant details. “/” means not mentioned in the article.CCL5: C-C Motif Chemokine Ligand 5; CCR5: C-C Chemokine Receptor Type 5; HSV-1: Herpes Simplex Virus Type 1; DRG: Dorsal Root Ganglion; TLR4: Toll-Like Receptor 4; TNF: Tumor Necrosis Factor; PHP: Pseudohypoparathyroidism; K+: Potassium ion; COX: Cyclooxygenase; PGE2: Prostaglandin E2; EP3: Prostaglandin E2 Receptor EP3; NMDA: N-Methyl-D-Aspartate; NR2Bs: N-Methyl-D-Aspartate Receptor Subunit 2B; NOS: Nitric Oxide Synthase; Prmt6: Protein Arginine Methyltransferase 6; cGAS: Cyclic GMP-AMP Synthase; STING: Stimulator of Interferon Genes; VPM: Ventral Posterior Medial Nucleus; VPL: Ventral Posterior Lateral Nucleus; P2X7: Purinergic Receptor P2X, Ligand-Gated Ion Channel 7; BBG: Brilliant Blue G; ER stress: Endoplasmic Reticulum stress; KCNA2: Potassium Voltage-Gated Channel Subfamily A Member 2; STAT3: Signal Transducer and Activator of Transcription 3; pSTAT3: Phosphorylated Signal Transducer and Activator of Transcription 3; MRC-5: Medical Research Council cell strain 5; TRPV1: Transient Receptor Potential Vanilloid 1; NO: Nitric Oxide; vHPPE: Ventriculoperitoneal hydrocephalus; VZV: Varicella-Zoster Virus; SNT: Syntrophin; H3: Histone H3; PWL: Paw Withdrawal Latency; MWT: Mechanical Withdrawal Threshold; TRX: Thioredoxin; NP: Nucleoside Phosphorylase; DNF: Brain-Derived Neurotrophic Factor; ASIC3: Acid-Sensing Ion Channel 3; PAQR: Progestin and AdipoQ Receptor; VGAT: Vesicular GABA Transporter; ERK: Extracellular Signal-Regulated Kinase; IE4: Immediate Early Protein 4

**Reference**

1. Wu S, Yang S, Li R, Ba X, Jiang C, Xiong D, et al. HSV-1 infection-induced herpetic neuralgia involves a CCL5/CCR5-mediated inflammation mechanism. J Med Virol. 2023;95: 1–16. doi:10.1002/jmv.28718

2. Silva CR, Melo BMS, Silva JR, Lopes AH, Pereira JA, Cecilio NT, et al. S100A9 plays a pivotal role in a mouse model of herpetic neuralgia via TLR4/TNF pathway. Brain Behav Immun. 2020;88: 353–362. doi:10.1016/j.bbi.2020.03.033

3. Ueda Y, Uta D, Tanbo S, Kawabata A, Kanayama S, Osaki M, et al. Inhibitory effect of amenamevir on acute herpetic pain and postherpetic neuralgia in mice infected with herpes simplex virus-1. J Dermatol Sci. 2020;98: 50–57. doi:10.1016/j.jdermsci.2020.03.004

4. Ichiro Takasaki, Ryota Nagashima, Takahiro Ueda, Tomoki Ogata, Arata Inoue, Kimiyasu Shiraki, Yoshimi Kitada SA. Fosphenytoin Alleviates Herpes Simplex Virus Infection-Induced Provoked and Spontaneous Pain-Like Behaviors in Mice. Biol Pharm Bull. 2022;45: 360–363.

5. Silva JR, Lopes AH, Talbot J, Cecilio NT, Rossato MF, Silva RL, et al. Neuroimmune–glia interactions in the sensory ganglia account for the development of acute herpetic neuralgia. J Neurosci. 2017;37: 6408–6422. doi:10.1523/JNEUROSCI.2233-16.2017

6. Takasaki I, Nojima H, Shiraki K, Sugimoto Y, Ichikawa A, Ushikubi F, et al. Involvement of cyclooxygenase-2 and EP3 prostaglandin receptor in acute herpetic but not postherpetic pain in mice. Neuropharmacology. 2005;49: 283–292. doi:10.1016/j.neuropharm.2004.12.025

7. Takasaki I, Taniguchi K, Komatsu F, Sasaki A, Andoh T, Nojima H, et al. Contribution of spinal galectin-3 to acute herpetic allodynia in mice. Pain. 2012;153: 585–592. doi:10.1016/j.pain.2011.11.022

8. Takasaki I, Andoh T, Nojima H, Shiraki K, Kuraishi Y. Gabapentin antinociception in mice with acute herpetic pain induced by herpes simplex virus infection. J Pharmacol Exp Ther. 2001;296: 270–275.

9. Kuraishi Y, Takasaki I, Nojima H, Shiraki K, Takahata H. Effects of the suppression of acute herpetic pain by gabapentin and amitriptyline on the incidence of delayed postherpetic pain in mice. Life Sci. 2004;74: 2619–2626. doi:10.1016/j.lfs.2004.01.005

10. Sasaki A, Nakashima Y, Takasaki I, Andoh T, Shiraki K, Kuraishi Y. Effects of loperamide on mechanical allodynia induced by herpes simplex virus type-1 in mice. J Pharmacol Sci. 2007;104: 218–224. doi:10.1254/jphs.FP0070294

11. Unezaki S, Sasaki A, Mabuchi T, Matsumura S, Katano T, Nakazawa T, et al. Involvement of Tyr1472 phosphorylation of NMDA receptor NR2B subunit in postherpetic neuralgia in model mice. Mol Pain. 2012;8: 1. doi:10.1186/1744-8069-8-59

12. Sasaki A, Serizawa K, Andoh T, Shiraki K, Takahata H, Kuraishi Y. Pharmacological differences between static and dynamic allodynia in mice with herpetic or postherpetic pain. J Pharmacol Sci. 2008;108: 266–273. doi:10.1254/jphs.08154FP

13. Nishikawa Y, Sasaki A, Andoh T, Nojima H, Shiraki K, Kuraishi Y. Modality-specific hyperexcitability of dorsal horn neurons to mechanical stimuli in herpetic mice. Neuroreport. 2009;20: 1077–1080. doi:10.1097/WNR.0b013e32832e0cc1

14. Asano K, Asahina S, Sakai M, Matsuda T, Ou K, Maeda Y, et al. Attenuating effect of artemin on herpes-related pain responses in mice infected with herpes simplex. In Vivo (Brooklyn). 2006;20: 533–538.

15. Sasaki A, Takasaki I, Andoh T, Nojima H, Shiraki K, Kuraishi Y. Roles of α-adrenoceptors and sympathetic nerve in acute herpetic pain induced by herpes simplex virus inoculation in mice. J Pharmacol Sci. 2003;92: 329–336. doi:10.1254/jphs.92.329

16. Sato-Takeda M, Takasaki I, Takeda K, Sasaki A, Andoh T, Nojima H, et al. Major histocompatibility complex haplotype is associated with postherpetic pain in mice. Anesthesiology. 2006;104: 1063–1069. doi:10.1097/00000542-200605000-00024

17. Asano K, Sameshima T, Shirasawa H, Hisamitsu T. Attenuating effect of mexiletine hydrochloride on herpetic pain in mice infected with herpes simplex virus. J Pharm Pharmacol. 2010;55: 1365–1370. doi:10.1211/0022357021828

18. Sasaki A, Mabuchi T, Serizawa K, Takasaki I, Andoh T, Shiraki K, et al. Different roles of nitric oxide synthase-1 and -2 between herpetic and postherpetic allodynia in mice. Neuroscience. 2007;150: 459–466. doi:10.1016/j.neuroscience.2007.09.067

19. Takasaki I, Sasaki A, Andoh T, Nojima H, Shiraki K, Kuraishi Y. Effects of analgesics on delayed postherpetic pain in mice. Anesthesiology. 2002;96: 1168–1174. doi:10.1097/00000542-200205000-00021

20. Takasaki I, Andoh T, Shiraki K, Kuraishi Y. Allodynia and hyperalgesia induced by herpes simplex virus type-1 infection in mice. Pain. 2000;86: 95–101. doi:10.1016/S0304-3959(00)00240-2

21. Kong E, Hua T, Li J, Li Y, Yang M, Ding R, et al. HSV-1 reactivation results in post-herpetic neuralgia by upregulating Prmt6 and inhibiting cGAS-STING. Brain. 2024.

22. Kramer PR, Strand J, Stinson C, Bellinger LL, Kinchington PR, Yee MB, et al. Role for the ventral posterior medial/posterior lateral thalamus and anterior cingulate cortex in affective/motivation pain induced by varicella zoster virus. Front Integr Neurosci. 2017;11: 1–12. doi:10.3389/fnint.2017.00027

23. Zhu Y, Zhang S, Wu Y, Wang J. P2X7 receptor antagonist BBG inhibits endoplasmic reticulum stress and pyroptosis to alleviate postherpetic neuralgia. Mol Cell Biochem. 2021;476: 3461–3468. doi:10.1007/s11010-021-04169-3

24. Kong C, Du J, Bu H, Huang C, Xu F, Ren H. LncRNA KCNA2-AS regulates spinal astrocyte activation through STAT3 to affect postherpetic neuralgia. Mol Med. 2020;26. doi:10.1186/s10020-020-00232-9

25. Garry EM, Delaney A, Anderson HA, Sirinathsinghji EC, Clapp RH, Martin WJ, et al. Varicella zoster virus induces neuropathic changes in rat dorsal root ganglia and behavioral reflex sensitisation that is attenuated by gabapentin or sodium channel blocking drugs. Pain. 2005;118: 97–111. doi:10.1016/j.pain.2005.08.003

26. Wallace VCJ, Segerdahl AR, Lambert DM, Vandevoorde S, Blackbeard J, Pheby T, et al. The effect of the palmitoylethanolamide analogue, palmitoylallylamide (L-29) on pain behaviour in rodent models of neuropathy. Br J Pharmacol. 2007;151: 1117–1128. doi:10.1038/sj.bjp.0707326

27. Cabrera JR, Viejo-Borbolla A, Alcamí A, Wandosell F. Secreted herpes simplex virus-2 glycoprotein G alters thermal pain sensitivity by modifying NGF effects on TRPV1. J Neuroinflammation. 2016;13: 1–9. doi:10.1186/s12974-016-0677-5

28. Zhang GH, Lv MM, Wang S, Chen L, Qian NS, Tang Y, et al. Spinal astrocytic activation is involved in a virally-induced rat model of neuropathic pain. PLoS One. 2011;6. doi:10.1371/journal.pone.0023059

29. Guedon JMG, Zhang M, Glorioso JC, Goins WF, Kinchington PR. Relief of pain induced by varicella-zoster virus in a rat model of post-herpetic neuralgia using a herpes simplex virus vector expressing enkephalin. Gene Ther. 2014;21: 694–702. doi:10.1038/gt.2014.43

30. Hasnie FS, Breuer J, Parker S, Wallace V, Blackbeard J, Lever I, et al. Further characterization of a rat model of varicella zoster virus-associated pain: Relationship between mechanical hypersensitivity and anxiety-related behavior, and the influence of analgesic drugs. Neuroscience. 2007;144: 1495–1508. doi:10.1016/j.neuroscience.2006.11.029

31. Guedon JMG, Yee MB, Zhang M, Harvey SAK, Goins WF, Kinchington PR. Neuronal changes induced by Varicella Zoster Virus in a rat model of postherpetic neuralgia. Virology. 2015;482: 167–180. doi:10.1016/j.virol.2015.03.046

32. Blackbeard J, Wallace VCJ, O’Dea KP, Hasnie F, Segerdahl A, Pheby T, et al. The correlation between pain-related behaviour and spinal microgliosis in four distinct models of peripheral neuropathy. Eur J Pain (United Kingdom). 2012;16: 1357–1367. doi:10.1002/j.1532-2149.2012.00140.x

33. Medhurst SJ, Collins SD, Billinton A, Bingham S, Dalziel RG, Brass A, et al. Novel histamine H3 receptor antagonists GSK189254 and GSK334429 are efficacious in surgically-induced and virally-induced rat models of neuropathic pain. Pain. 2008;138: 61–69. doi:10.1016/j.pain.2007.11.006

34. Stinson C, Logan SM, Bellinger LL, Rao M, Kinchington PR, Kramer PR. Estradiol Acts in Lateral Thalamic Region to Attenuate Varicella Zoster Virus Associated Affective Pain. Neuroscience. 2019;414: 99–111. doi:10.1016/j.neuroscience.2019.06.029

35. Wang Z, Shen W, Zhu M, Xu M, Qiu M, Zhang D, et al. MiR-199-3p Suppressed Inflammatory Response by Targeting MECP2 to Alleviate TRX-Induced PHN in Mice. Cell Transplant. 2022;31. doi:10.1177/09636897221108192

36. Hong B, Sun J, Zheng H, Le Q, Wang C, Bai K, et al. Effect of tetrodotoxin pellets in a rat model of postherpetic neuralgia. Mar Drugs. 2018;16: 1–14. doi:10.3390/md16060195

37. Wei X, Wang L, Hua J, Jin X hong, Ji F, Peng K, et al. Inhibiting BDNF/TrkB.T1 receptor improves resiniferatoxin-induced postherpetic neuralgia through decreasing ASIC3 signaling in dorsal root ganglia. J Neuroinflammation. 2021;18: 1–17. doi:10.1186/s12974-021-02148-5

38. Stinson C, Deng M, Yee MB, Bellinger LL, Kinchington PR, Kramer PR. Sex differences underlying orofacial varicella zoster associated pain in rats. BMC Neurol. 2017;17: 1–15. doi:10.1186/s12883-017-0882-6

39. Hornung RS, Kinchington PR, Umorin M, Kramer PR. PAQR8 and PAQR9 expression is altered in the ventral tegmental area of aged rats infected with varicella zoster virus. Mol Pain. 2023;19: 1–10. doi:10.1177/17448069231202598

40. Kramer P, Rao M, Stinson C, Bellinger LL, Kinchington PR, Yee MB. Aromatase derived estradiol within the thalamus modulates pain induced by varicella zoster virus. Front Integr Neurosci. 2018;12: 1–14. doi:10.3389/fnint.2018.00046

41. Warner BE, Yee MB, Zhang M, Hornung RS, Kaufer BB, Visalli RJ, et al. Varicella-zoster virus early infection but not complete replication is required for the induction of chronic hypersensitivity in rat models of postherpetic neuralgia. PLoS Pathogens. 2021. doi:10.1371/journal.ppat.1009689

42. Hornung R, Pritchard A, Kinchington PR, Kramer PR. Reduced activity of GAD67 expressing cells in the reticular thalamus enhance thalamic excitatory activity and varicella zoster virus associated pain. Neurosci Lett. 2020;736: 135287. doi:10.1016/j.neulet.2020.135287

43. Kramer PR, Stinson C, Umorin M, Deng M, Rao M, Bellinger LL, et al. Lateral thalamic control of nociceptive response after whisker pad injection of varicella zoster virus. Neuroscience. 2017;356: 207–216. doi:10.1016/j.neuroscience.2017.05.030

44. Fleetwood-Walker SM, Quinn JP, Wallace C, Blackburn-Munro G, Kelly BG, Fiskerstrand CE, et al. Behavioural changes in the rat following infection with varicella-zoster virus. J Gen Virol. 1999;80: 2433–2436. doi:10.1099/0022-1317-80-9-2433

45. Sadzot‐Delvaux C, Merville‐Louis MP, Delree P, Marc P, Piette J, Moonen G, et al. An in vivo model of varicella‐zoster virus latent infection of dorsal root ganglia. J Neurosci Res. 1990;26: 83–89. doi:10.1002/jnr.490260110
